# Haplotype-resolved genome of a dry-preservable cell line reveals chromosome evolution and DNA damage

**DOI:** 10.26508/lsa.202603719

**Published:** 2026-10-06

**Authors:** Yuki Yoshida, Takahiro Kikawada

**Affiliations:** 1 Extremotolerance Biotechnology Group, Department of Biomaterial Sciences, Institute of Agrobiological Sciences (NIAS), National Agriculture and Food Research Organization (NARO) , Ibaraki, Japan; 2 Department of Integrated Biosciences, Graduate School of Frontier Sciences, The University of Tokyo , Tokyo, Japan

## Abstract

Anhydrobiosis, the ability to survive extreme desiccation, occurs in diverse organisms and provides a framework for understanding how cells preserve genome integrity under severe water loss. In insects, this phenotype is rare, and mechanistic studies have focused on the chironomid midge *Polypedilum vanderplanki* and its cultured cell line, Pv11. Previous genomic resources for this system were limited by the use of mixed insect and cell-line DNA. Here, we generated a high-quality chromosome-level, haplotype-resolved assembly from a clonal Pv11 line. Chromosome 4 showed markedly elevated heterozygosity and haplotype-specific genes. Comparative gene synteny with other chironomids revealed extensive intrachromosomal rearrangement, but largely conserved broad chromosome identity across subfamily lineages. Finally, genome-wide mapping of DNA double-strand breaks by sBLISS showed that breaks during trehalose pretreatment accumulate preferentially near highly expressed transcription start sites. Together, these results provide a haplotype-resolved genomic framework for linking chromosome evolution, regulatory chromatin, and genome integrity during entry into anhydrobiosis.

## Abstract (precis)

This study establishes a diploid genome resource for desiccation-tolerant insect cells, enabling new insights into genome organization and stress-associated DNA damage.

## Introduction

Small-bodied animals with limited dispersal capacity are especially susceptible to rapid or recurrent environmental fluctuations. Among the most severe stressors is desiccation, in which aquatic or semiaquatic organisms experience extensive water loss during prolonged arid conditions. Severe dehydration disrupts cellular homeostasis and damages biomolecules, frequently resulting in mortality. Nonetheless, several invertebrate lineages have evolved the ability to survive near-complete dehydration, a phenomenon termed anhydrobiosis (Keilin, 1959).

Our research focuses on anhydrobiosis in larvae of the African chironomid *Polypedilum vanderplanki* (Diptera; Chironomidae) (Hinton, 1960). Together with its closest relative, *Polypedilum pembai* (Cornette et al, 2017), it represents the only known anhydrobiotic lineage within Insecta. Dipteran models have enabled powerful genetic and genomic analyses across diverse biological questions, providing a strong comparative framework for mechanistic studies. A distinctive advantage of *Po. vanderplanki* is the availability of an anhydrobiosis-capable cultured cell line, Pv11 (Nakahara et al, 2010), derived from its embryos; comparable cell systems are not available for other anhydrobiotic animals such as tardigrades, rotifers, or nematodes. Exposing Pv11 cells to 600 mM trehalose for 48 h induces anhydrobiosis-associated gene expression, enabling ∼20% of cells to survive several months in a desiccated state at room temperature and revive upon rehydration (Watanabe et al, 2016). Pv11 cells therefore offer a tractable platform for dissecting the molecular basis of anhydrobiosis under controlled experimental conditions.

A chromosome-level genome assembly of *Po. vanderplanki* was previously reported, and chromosome 4 (Chr4) was proposed to function as a “sandbox” for adaptive genetic modifications (Yoshida et al, 2022b). However, elevated heterozygosity on Chr4, together with limitations of earlier sequencing technologies, produced haplotype switching, consensus errors, and local misassemblies, which in turn reduced confidence in single-nucleotide variant (SNV) detection. In addition, the prior reference merged DNA derived from the insect and the Pv11 cell line, potentially introducing bias because cultured cells can diverge from their source genomes. More accurate haplotype-level analyses and a stronger foundation for Pv11 genomics therefore require a haplotype-resolved diploid assembly generated directly from a clonal Pv11 cell line.

To address these limitations, we generated high-fidelity (HiFi) PacBio data from a clonal Pv11 cell line and assembled a haplotype-resolved diploid genome, enabling systematic identification of sequence variants between the two haplotypes. Using this assembly, we investigated chromosome evolution through comparative genomic analyses and mapped DNA double-strand breaks (DSBs) during trehalose pretreatment to examine their relationship with transcriptional activity.

## Results

### Assembly of a high-quality diploid Pv11 genome

Reassembly of previously generated PacBio CLR data (Table S1) was first evaluated to determine whether recent assembly algorithms improve genome quality. NextDenovo produced a 117 Mb assembly comprising 60 contigs (v6.0), with 96.44% BUSCO completeness (Diptera lineage) and a Merqury QV of 39.17 (Table S2). Hi-C–based scaffolding generated a chromosome-scale assembly (v6.1), after which diploid reconstruction was attempted by phasing SNVs using Hi-C data (v6.2). This phasing-based approach reduced consensus quality, as reflected by a lower Merqury QV than v6.0, indicating that higher accuracy and/or higher coverage long reads were required for robust haplotype resolution.

Table S1. Sequence data statistics. Details of the cell lines and sequencing data used in this study.

Table S2. Genome assembly statistics. Assembly statistics for previously published *Po. vanderplanki* genomes and the genome assemblies generated in this study.

**Figure 1.**
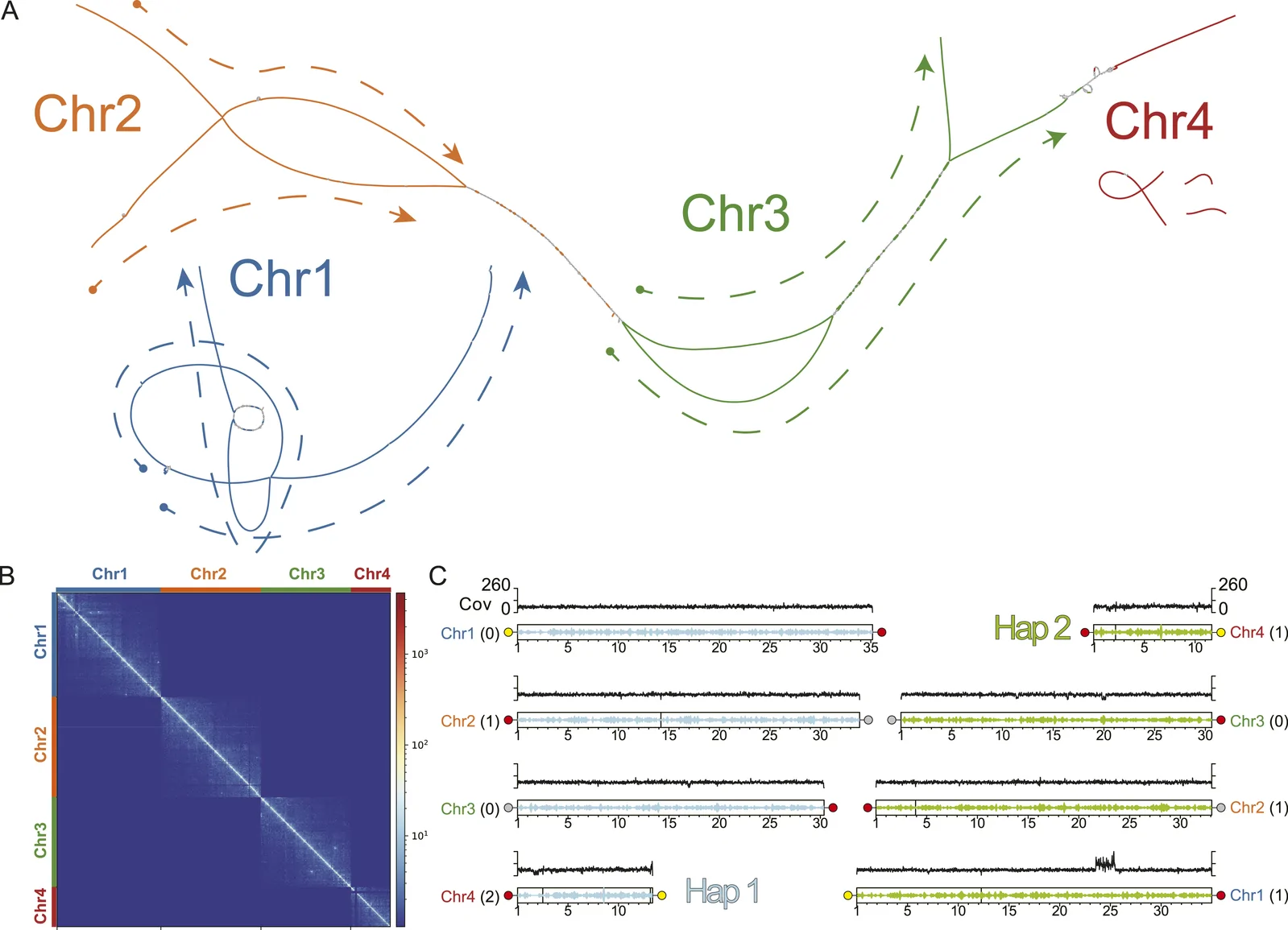
Diploid genome of the anhydrobiotic Pv11 cell line. **(A)** PacBio CCS and Hi-C reads were assembled with hifiasm, and the assembly graph was visualized with Bandage. Contigs were annotated by mapping to Pv11_5.2 with minimap2; arrows indicate contig orientation (5′–3′). **(B)** Genome-wide Hi-C contact map for the haplotype 1 chromosome-scale assembly. Chromosome identities are indicated by colored bars corresponding to Chr1–Chr4. **(C)** Karyotype plots for both haplotypes. For each chromosome, assembly gaps (vertical black lines within the gene density bar plot; counts are shown in parentheses next to chromosome labels), HiFi read coverage (10-kb bins; black lines), and gene density (blue and green bars) are shown. Colored dots at chromosome ends indicate candidate telomeric repeat units (Unit_A, red; Unit_B, yellow; neither detected, gray).

Accordingly, 14.8 Gb of PacBio HiFi sequencing data was generated from a clonal Pv11 single-cell isolation B cell (Pv11 SCI-B) (Table S1). HiFi reads were assembled with hifiasm and integrated with previously published Hi-C data (Yoshida et al, 2022b) and ONT long reads (Miyata et al, 2022), yielding 166 haplotype-resolved raw contigs (Fig 1A, statistics shown in Table S2). Haplotype-aware scaffolding produced eight chromosome-level contigs matching lengths obtained in our previous assembly Pv11_5.2, and 152 unplaced contigs. Error correction with T2T-Polish generated the final v7.0 assembly (Table S2), which achieved a Merqury QV of 60.52 and a compleasm completeness of 96.62% (Diptera lineage), representing a substantial improvement over earlier versions. Repetitive sequences accounted for ∼16% of the genome.

**Figure S1.**
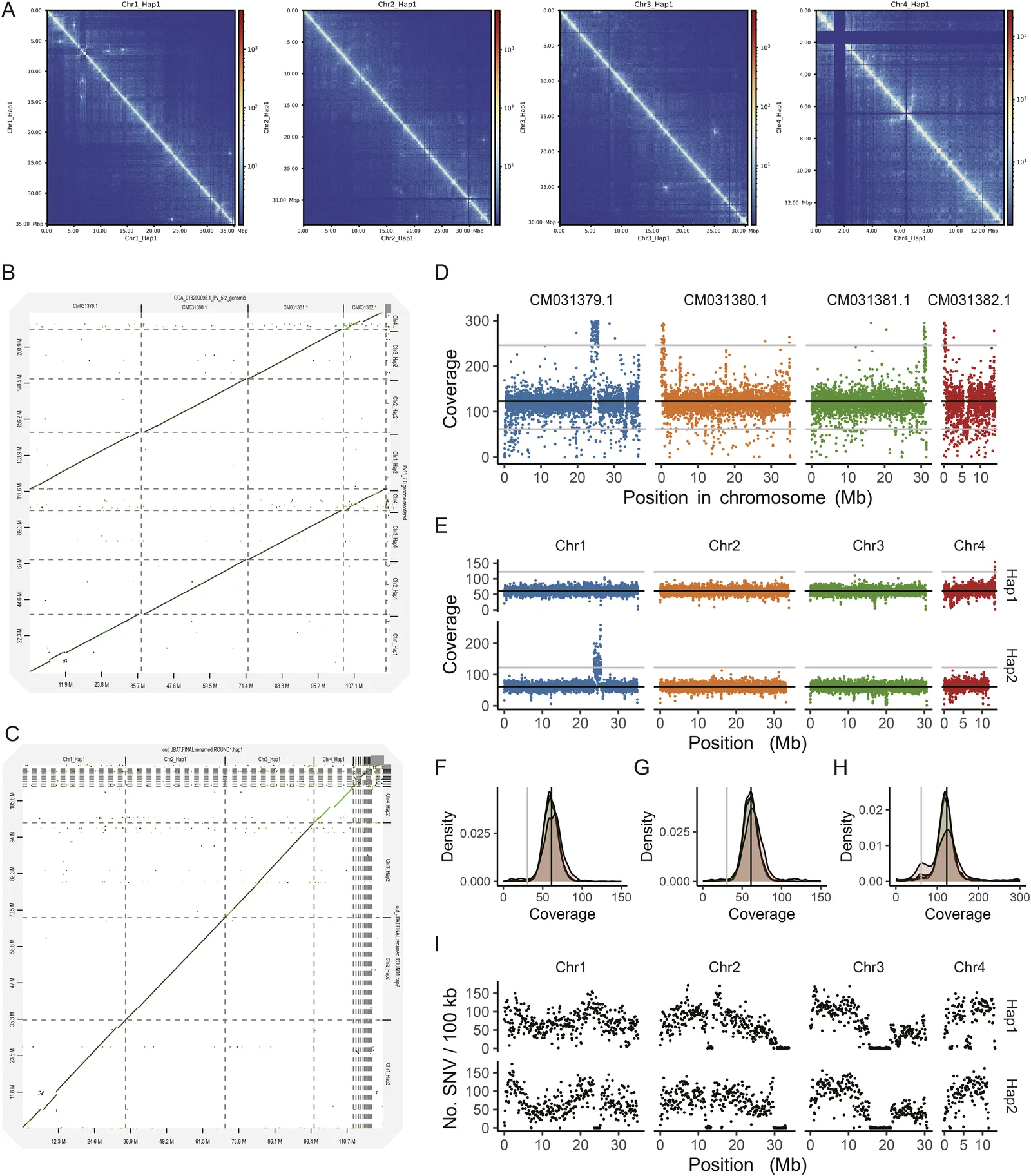
Assembly and quality assessment of the Pv11 diploid genome. **(A)** Hi-C contact map for haplotype 1 chromosomes. Hi-C contact matrices were generated by mapping Hi-C reads to the diploid genome and visualized for haplotype 1. **(B)** Alignment of the v7.0 assembly to the previous v5.2 reference. Hap1 and Hap2 assemblies were aligned to v5.2 using D-GENIES; chromosome boundaries are indicated by dashed black lines. **(C)** Direct haplotype-to-haplotype alignment. Hap1 and Hap2 assemblies were aligned using D-GENIES; chromosome boundaries are indicated by dashed black lines. **(D, E)** Genome-wide coverage distributions of PacBio HiFi reads mapped to v5.2 and v7.0, respectively. The black line indicates the genome-wide mean coverage; gray lines indicate 0.5× and 2× coverage thresholds. **(F, G, H)** Per-bin (10 kb) coverage distributions for haplotype 1, haplotype 2, and v5.2, respectively. A secondary peak at approximately half of the genome-wide mean is observed for v5.2. **(I)** SNVs detected from insect genomic DNA. Illumina short reads were mapped to the diploid genome, SNVs were called, and counts were summarized in 100-kb windows.

The chromosome-scale scaffolds contained six gaps in total (Hap1: Chr1 = 0, Chr2 = 1, Chr3 = 0, Chr4 = 2; Hap2: Chr1 = 1, Chr2 = 1, Chr3 = 0, Chr4 = 1). The Hi-C contact map for haplotype 1 showed a weak plaid-like pattern consistent with chromatin compartmentalization (Figs 1B and S1A). Notably, both Chr4 haplotypes contained a gap at a corresponding position (Hap1: 2,496,276; Hap2: 2,159,598). Inspection of the flanking sequence by Tandem Repeats Finder revealed a highly ordered array of a 21-mer motif (5′-TTC​CAC​ATT​TAT​TCC​AAC​TTA-3′). High-copy arrays of this motif were detected only on Chr3_Hap2 and on both Chr4 haplotypes, suggesting it is unlikely to be the primary centromeric repeat.

Alignment to the previous reference assembly (Pv11_5.2) identified a haplotype-specific intrachromosomal translocation on Chr1 and haplotype-specific deletions on Chr1 and Chr4 (Fig S1B and C). Consistent with improved consensus accuracy, HiFi read mapping showed more uniform coverage for v7.0 than for v5.2 (Fig S1D–H). In addition, a 2- to 3-Mb duplication was detected on Chr1 of haplotype 2 (Fig 1C). Because cultured cell lines can accumulate mutations during long-term propagation, we quantified SNVs that distinguish Pv11 from its parental *Po. vanderplanki* genome. In total, 153,945 SNVs were detected, with uneven accumulation across chromosomes (Fig S1I and Table S3).

Table S3. Insect GATK. SnpEff annotations of SNVs detected by GATK from insect gDNA-seq data.

Gene prediction with BRAKER3 identified 30,930 genes (Hap1: 15,790; Hap2: 15,140), with 97.42% compleasm completeness (Hap1: 96.13%; Hap2: 95.74%). Slightly lower compleasm completeness for individual haplotypes suggested the presence of haplotype-specific BUSCO genes. We also identified 128 genes with strong evidence of horizontal gene transfer (Hap1: 65; Hap2: 63; 0.41% of predicted genes), including 35 located on Chr4 (Hap1: 19; Hap2: 16; 0.11% of predicted genes). This was relatively consistent with our previous estimation of 70 horizontally transferred genes (Yoshida et al, 2022b).

### Chr4 shows elevated heterozygosity and coding-sequence divergence, particularly among lowly expressed genes

**Figure 2.**
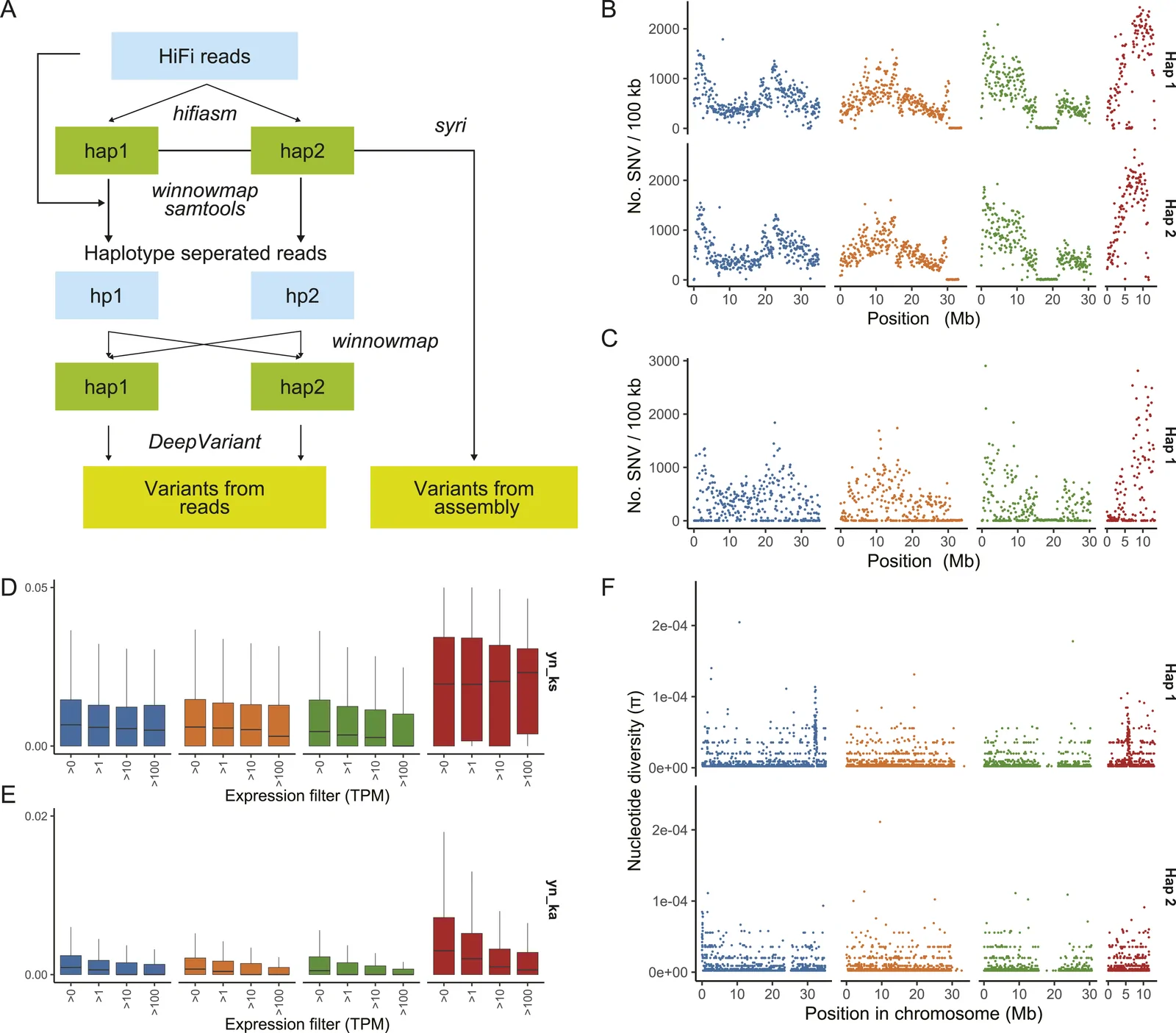
High heterozygosity on Chr4 and its impact on coding sequences. **(A)** SNV detection workflow. PacBio CCS reads were haplotype-partitioned by mapping to the diploid genome, and variants were called with DeepVariant; variants were also inferred by direct haplotype alignment using SyRI. **(B)** Heterozygosity from haplotype-separated CCS read mapping (DeepVariant). **(C)** Heterozygosity from haplotype-to-haplotype alignment (SyRI). **(D, E)** Ks and Ka distributions for haplotype ortholog pairs (MCScan), respectively. **(F)** Nucleotide diversity among independently cultured Pv11 populations (short-read SNVs; VCFtools).

Elevated heterozygosity on Chr4 was previously reported, raising the possibility that this chromosome serves as a hotspot for divergence. To confirm that the signal was not an assembly artifact, base-level variation between haplotypes was reestimated using two independent approaches (Fig 2A). First, haplotype-separated HiFi reads were mapped to the diploid assembly and SNVs were called with DeepVariant (Fig 2B). Second, SNVs were inferred by direct haplotype-to-haplotype alignment using SyRI (Fig 2C). Both methods consistently showed increased SNV density per 100 kb on Chr4, supporting the earlier conclusion. The higher heterozygosity estimated by SyRI likely reflects methodological differences between read-based variant calling and contig alignment.

Annotation of SyRI-derived SNVs relative to gene features (Table S4) indicated that Chr1–3 were dominated by intergenic (34–36%), intronic (18–19%), and upstream/downstream (16–18%) variants. Chr4 showed a distinct distribution, with enrichment in upstream (25%) and downstream (26%) regions and reduced intergenic (27%) and intronic (13%) fractions. HOMER annotatePeaks confirmed these trends, with the exception of intronic variants, which showed significant enrichment (Table S5). Randomly simulated variants did not reproduce these patterns (Table S5). Although the number of exonic SNVs was similar across chromosomes, Chr4 contained more missense variants, consistent with earlier analyses. Together, these results suggest that Chr4 variation disproportionately affects gene-proximal and coding regions, although the mechanisms underlying this pattern remain unresolved.

Table S4. SyRI SNVs. SnpEff annotations of SNVs detected by SyRI through haplotype comparison.

Table S5. Random SnpEff. SnpEff annotations of simulated SNVs generated from randomly sampled genomic positions.

To assess divergence at the gene level, gene models were lifted over between haplotypes and compared with prior haploid assemblies (v5.2 and v6.0). Only 40% of predicted genes encoded identical amino acid sequences between haplotypes (Table S6), with the lowest identity observed on Chr4. In addition, 6.6% of Hap1 genes and 2.9% of Hap2 genes were not recovered in the opposite haplotype. Approximately 20–25% of these haplotype-specific genes mapped to Chr4 (25.9% for haplotype 1 and 21.7% for haplotype 2), although Chr4 spans only 11.9% of the genome and harbors 14.3% of the transcripts.

Table S6. Liftoff between haplotypes. Liftoff statistics and gene locations obtained from comparisons between genome versions and haplotypes.

Homologous gene pairs between haplotypes were then identified to estimate synonymous (Ks) and nonsynonymous (Ka) substitution rates (Fig 2D and E). Both Ka and Ks were elevated on Chr4, consistent with increased divergence. When stratified by expression, Ka values on Chr4 decreased to levels comparable to other chromosomes, whereas Ks remained high. This pattern is consistent with purifying selection acting more strongly on highly expressed genes to remove amino acid–altering substitutions, whereas synonymous changes accumulate.

### No evidence for ongoing Chr4-specific divergence across independently maintained Pv11 populations

The accumulation of synonymous and nonsynonymous substitutions on Chr4 raised the possibility that the two Chr4 haplotypes have diverged through independent drift, potentially altering gene content and function. This hypothesis was tested by quantifying genome-wide variation across independently maintained Pv11 populations, which had been independently maintained under broadly similar in vitro conditions.

**Figure S2.**
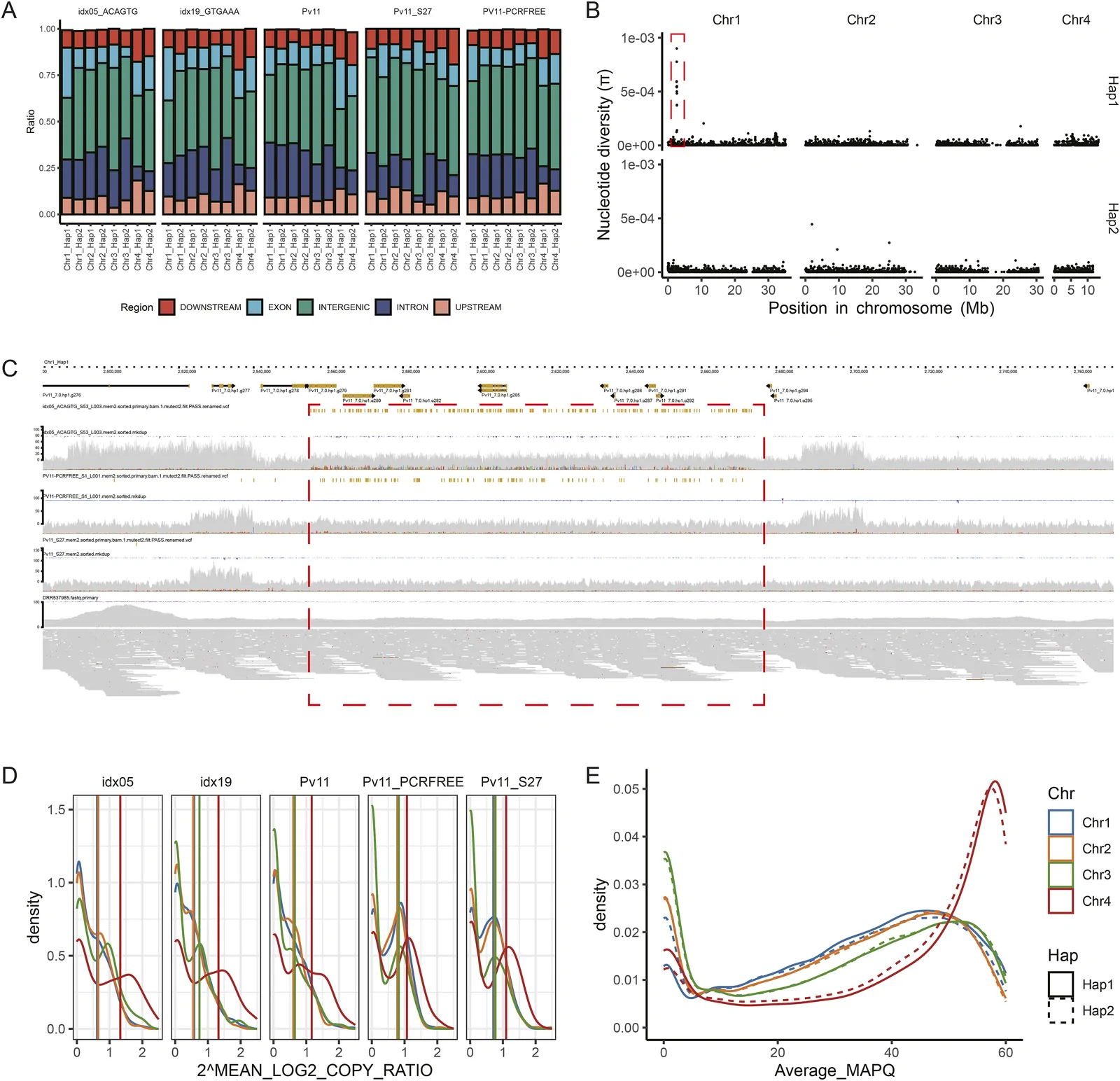
SNVs detected in Pv11 populations. **(A)** Annotation of population SNVs relative to gene features. SNVs were annotated using SnpEff and classified as upstream, downstream, exonic, intronic, or intergenic. **(B)** Genome-wide nucleotide diversity across Pv11 populations. Nucleotide diversity (π) was computed from population SNVs and plotted along the genome. The prominent Chr1 peak was excluded when generating Fig 2F. **(C)** JBrowse visualization of a highly variable region on Chr1. Tracks include gene models, SNVs, and read alignments from multiple populations (idx05, PCRFREE, Pv11_S27, and HiFi). The red box highlights a region with variants enriched in idx05 and PCRFREE. **(D)** Distribution of inferred copy-number ratios. Copy-number ratios were estimated using the GATK CNV workflow, and the distribution of 2^MEAN_LOG2_COPY_RATIO was plotted as a density curve. Colors denote chromosomes; vertical lines indicate chromosome-specific medians. **(E)** MAPQ distribution of Pv11_S27 reads. Short reads were mapped to the diploid assembly. Chr1–3 show broader MAPQ distributions consistent with higher ambiguity in highly similar regions, whereas Chr4 is strongly enriched near MAPQ 60.

GATK-based calling identified 556–8,446 SNVs per population (Table S7), with the most recently established culture (Pv11_S27) showing the fewest variants. Only 124 SNV sites were shared across all populations, whereas 10,679 were population-specific. Similar to the haplotype comparison, variants were enriched in upstream and downstream regions (Fig S2A). Genome-wide scans for elevated interpopulation diversity initially highlighted a variable interval on Chr1 (Chr1_Hap1: 2,473,867–2,722,845; Fig S2B). Visualization in JBrowse2 showed that SNVs in this region were concentrated in two populations (idx05 and PCRFREE; Fig S2C) and coincided with ∼2× read depth relative to flanking regions, suggesting copy-number variation (CNV) that was not supported by HiFi data.

Table S7. Population GATK. SnpEff annotations of SNVs detected by GATK from Pv11 gDNA-seq data across multiple sequencing datasets.

To evaluate the possibility of CNV, CNV profiling was performed using the GATK CNV pipeline with 1-kb windows and copy-ratio profiles were compared across chromosomes and Pv11 populations. This analysis indicated elevated copy ratios on Chr4 relative to Chr1–3 (Fig S2D).

Because Chr4 also exhibits unusually high heterozygosity, the possibility of a mapping-driven artifact was examined. Read mapping quality (MAPQ) was assessed for the most recent genomic DNA dataset (Pv11_S27) aligned to the diploid genome. Chr4 showed a pronounced MAPQ peak centered around 55–60, whereas Chr1–3 displayed broader MAPQ distributions (Fig S2E). One plausible explanation is that sequence divergence between homologous regions reduces ambiguous alignments, increasing MAPQ for reads originating from Chr4, whereas higher similarity between homologs on Chr1–3 increases multimapping ambiguity and lowers MAPQ. As the GATK CNV workflow filters low-quality alignments (e.g., reads with MAPQ < 30 in the read-counting step by default), chromosome-scale differences in MAPQ can bias read-depth estimates and inflate apparent copy ratios in more divergent regions. Taken together, these observations suggest that the elevated Chr4 copy ratios are more likely to reflect MAPQ-dependent read-counting bias associated with high heterozygosity than true CNV.

After excluding these problematic regions, nucleotide diversity was broadly uniform across the genome, except for two intervals on Chr1 and Chr4 (Fig 2F) that overlapped haplotype-specific regions and may therefore represent residual assembly-related effects.

Overall, despite substantial haplotype divergence on Chr4, population-level data did not support widespread, haplotype-specific genetic drift across independently cultured lines.

### Higher order repeats in the Pv11 genome

Chironomid telomeres consist of long tandem arrays of complex repeat units rather than the short telomerase-generated repeats found at chromosome ends in many eukaryotes (Saiga & Edstrom, 1985; Cohn & Edstrom, 1992). To identify candidate telomeric repeats in Pv11, we manually inspected the terminal regions of chromosome-scale scaffolds. We identified two repeat units, Unit_A (150 bp) and Unit_B (303 bp), which were detected at 12 of the 16 scaffold ends in the haplotype-resolved assembly (2n = 8; Fig 1C), but absent from the remaining four ends corresponding to one end of Chr2 and one end of Chr3 in each haplotype. Chr2 and Chr3 were also connected in the assembly graph, raising the possibility that these scaffold ends represent an unresolved Chr2-Chr3 junction. However, the genome-wide Hi-C map did not provide clear independent support for contact between the relevant Chr2 and Chr3 ends, and the available long reads did not resolve an unambiguous junction. Thus, the current data neither confirm nor exclude a Chr2-Chr3 fusion. Resolving this possibility would require additional ultra-long-read sequencing and cytogenetic validation.

Both repeat units were present in the v5.2 assembly but were largely absent from v0.9. Short reads used for the v0.9 assembly showed above-average coverage when mapped to these telomeric regions, suggesting that the apparent absence in v0.9 reflected limitations of short-read assembly rather than biological loss. Several terminal structures remained unresolved. For example, the telomeric end of Chr1_Hap2 was substantially shorter than that of Hap1, which contained an inverted-repeat structure.

Notably, Unit_A and Unit_B were not detected in available genomes of *Polypedilum nubifer*, *Po. pembai*, or *Chironomus riparius*, and no homologous sequences were identified in National Center for Biotechnology Information (NCBI). To further evaluate their specificity, we mapped *Po. nubifer* genomic DNA reads to the Pv11 haplotype 1 assembly; the repeat loci showed limited coverage, consistent with the absence of closely related sequences in *Po. nubifer*. However, interpretation is constrained by the fragmented, short-read–based assemblies for *Po. nubifer* and *Po. pembai*, potential biases from cross-species read mapping, and the phylogenetic distance of *C. riparius*. Fluorescence in situ hybridization would provide an orthogonal test of repeat localization and could facilitate detection of related telomeric elements in other chironomids. Finally, no definitive centromeric repeat units were identified by CentroAnno, suggesting that additional high-quality *Polypedilum* assemblies and/or cytogenetic mapping may be required to resolve centromeric features.

### Extensive intrachromosomal rearrangement across divergent chironomid subfamily lineages

**Figure 3.**
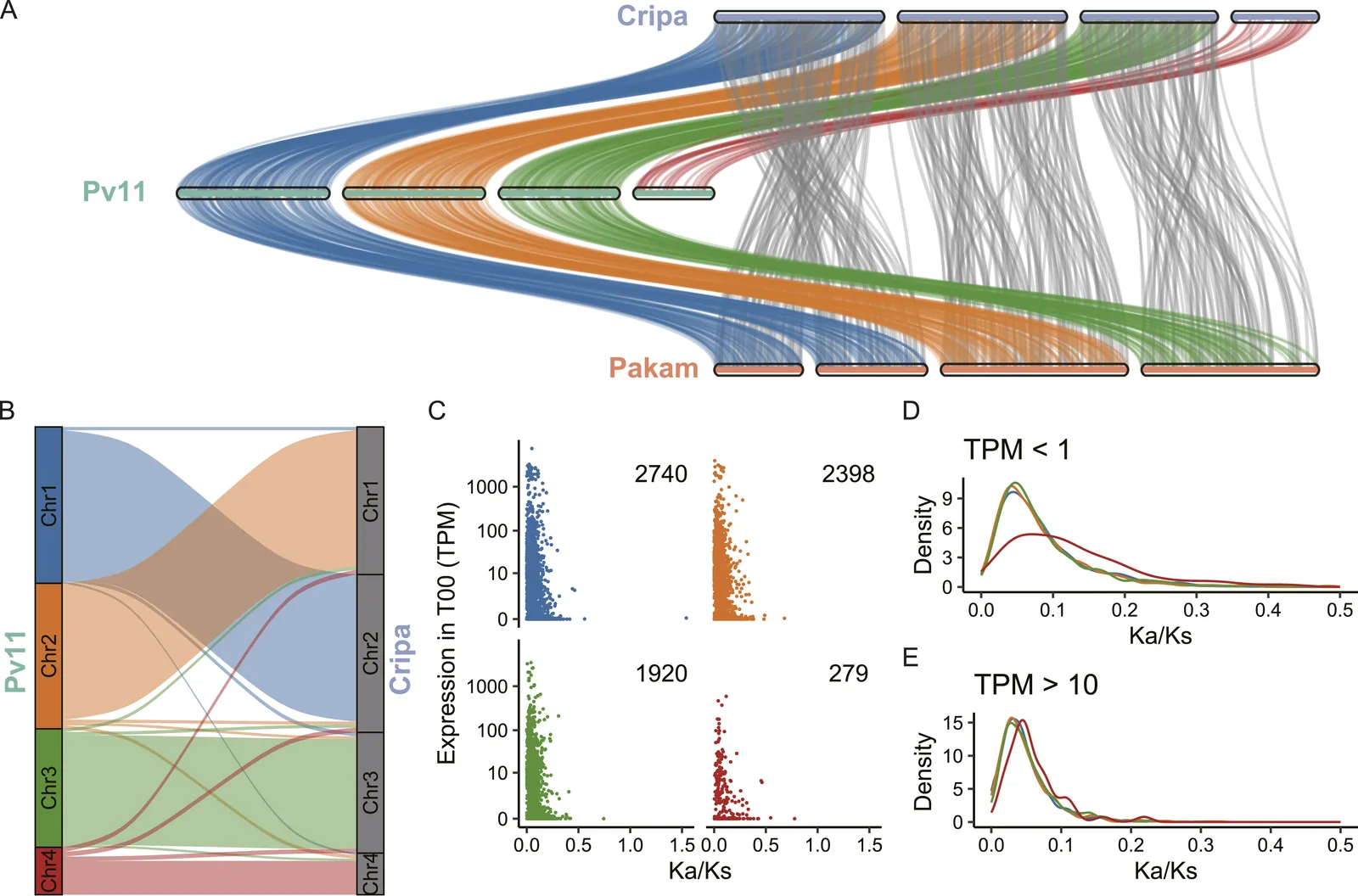
Comparative chromosome organization across divergent chironomid subfamily lineages. **(A)** Gene-level synteny analysis between Pv11 (v7.0 haplotype 1), *C. riparius* (Cripa), and *Pr. akamusi* (Pakam) reveals extensive intrachromosomal reshuffling across all chromosomes. The colors indicate chromosomes as defined in Fig 1. **(B)** Chromosomal distribution of bidirectional best-hit orthologs between Pv11 and *C. riparius*. **(C)** Relationship between Ka/Ks and gene expression at T00 (hydrated cells). **(D, E)** Ka/Ks distributions for genes with TPM < 1 and TPM > 10, respectively.

Cytogenetic analyses of polytene chromosomes in the salivary glands of chironomids have demonstrated extensive intrachromosomal rearrangement, possibly mediated by repetitive elements (Bovero et al, 2002; Kiknadze, 2008; Michailova et al, 2009). Taking advantage of the highly contiguous Pv11 genome assembly, we compared gene-level synteny with *C. riparius* from the same subfamily, Chironominae, and *Propsilocerus akamusi* from a different subfamily, Orthocladiinae sensu lato. Small synteny blocks were detected across all four chromosomes between Pv11 and *C. riparius* but were extensively rearranged within chromosomes (Fig 3A). Similarly fragmented synteny was observed in comparisons involving *Pr. akamusi*, including both Pv11-to-*Pr. akamusi* and *C. riparius*-to-*Pr. akamusi*. Thus, extensive intrachromosomal rearrangement was not restricted to the comparison between the two Chironominae species but was also evident in comparisons spanning a deeper subfamily-level divergence.

Chr4 contained fewer detectable blocks than the other chromosomes in the Pv11-to-*C. riparius* comparison and showed no detectable conserved blocks in the Pv11-to-*Pr. akamusi* comparison, consistent with our previous analyses (Yoshida et al, 2022b). Despite pervasive intrachromosomal reshuffling, bidirectional best-hit BLAST search between Pv11 and *C. riparius* assigned most orthologs to corresponding chromosomes in both species (Fig 3B), indicating that broad chromosome gene content has been retained even though local gene order has been extensively reorganized, with Chr4 showing the most pronounced structural divergence.

To examine selective constraints, Ka/Ks was estimated for synteny-defined ortholog pairs (Fig 3C). Genes with near-zero expression in hydrated cells (TPM < 1) showed elevated Ka/Ks, particularly on Chr4 (Fig 3D), consistent with relaxed purifying selection among lowly expressed genes. This trend was not evident among more highly expressed genes (TPM > 10; Fig 3E), suggesting that genes with minimal expression under conditions before the trehalose treatment (T00) may experience weaker selective constraint.

### DSBs are enriched around transcription start sites

**Figure 4.**
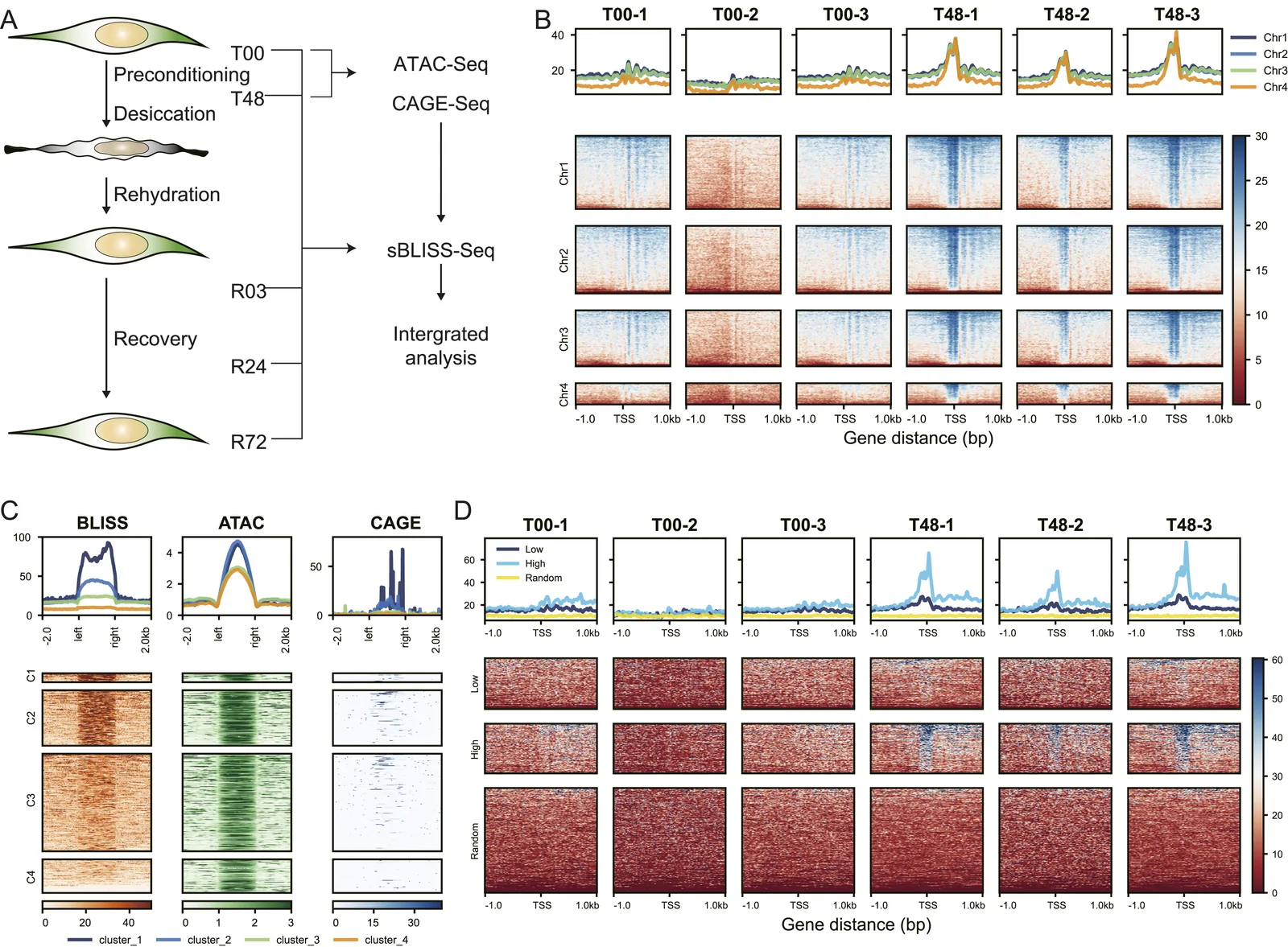
DNA DSBs are enriched near TSSs during induction of anhydrobiosis. **(A)** Experimental design and integration of sBLISS with ATAC-seq and CAGE-seq. Pv11 cells were collected before (T00) and after (T48) the 48-h trehalose pretreatment used to induce desiccation tolerance, and at 3 h (R3), 24 h (R24), and 72 h (R72) after rehydration of the desiccated cells. **(B)** Genome-wide DSB profiles around CAGE-defined TSSs. **(C)** Integrated sBLISS/ATAC-seq/CAGE-seq signals at ATAC peaks clustered by sBLISS profile. **(D)** DSB profiles around TSSs stratified by expression; randomized loci are negative controls.

The v7.0 assembly enabled genome-wide mapping of DNA DSBs across the Pv11 anhydrobiosis cycle. sBLISS was performed at two stages of trehalose pretreatment (T00 and T48) and at multiple post-rehydration time points (R03, R24, R72; Fig 4A).

**Figure S3.**
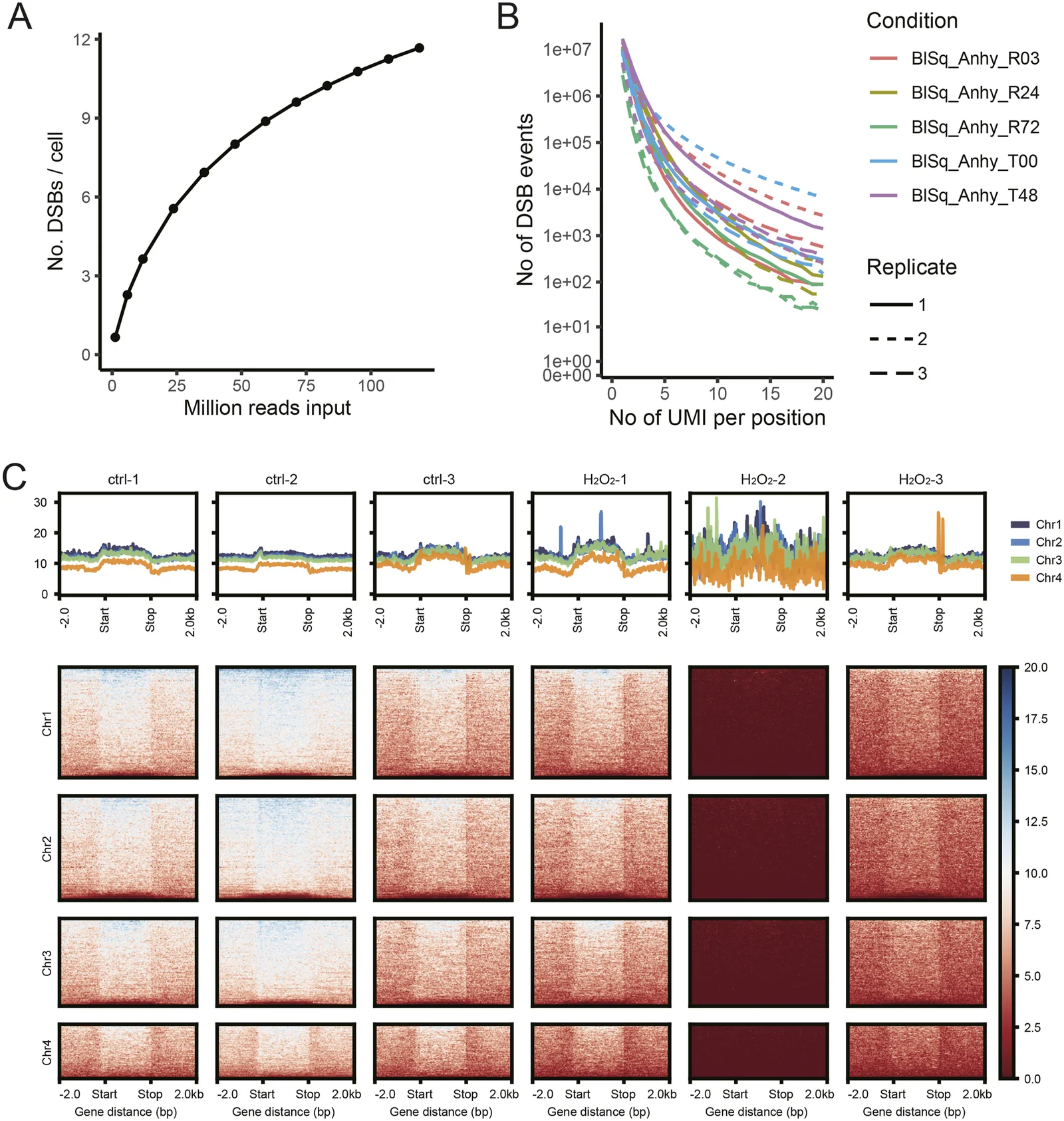
Summary of the sBLISS dataset. **(A)** DSB detection as a function of sequencing depth. Raw reads from the deepest library were subsampled, and DSB counts were recalculated at each depth. **(B)** Distribution of UMI counts per DSB event. **(C)** DSB profiles across gene features in H_2_O_2_-treated controls. H_2_O_2_-induced damage shows a more diffuse distribution than the TSS-enriched pattern observed in trehalose-treated samples.

In the original BLISS study, Yan and colleagues estimated that BLISS detects ∼80–100 DSBs per cell with ∼50 million input reads (Yan et al, 2017). To assess whether comparable sensitivity applies to Pv11, the R03-3 dataset (the deepest library) was subsampled and DSBs per cell were quantified as a function of sequencing depth. Even at >100 million reads, the estimated DSBs per cell did not reach a plateau (Fig S3A). The library with the greatest sequencing depth (193 million reads) yielded ∼12 detected DSBs per cell (Table S8), substantially fewer than reported by Yan et al (2017). Subsampling analysis indicated that this sequencing depth did not saturate the number of detected DSBs per cell, suggesting that deeper sequencing would be required to more comprehensively characterize the breakome of this genome.

Table S8. sBLISS subsampling. Number of DSBs detected in subsampled sBLISS datasets.

Several factors could contribute to this discrepancy. First, the Pv11 haploid genome (∼120 Mb) is much smaller than mammalian genomes (∼3 Gb), which changes mappability and the probability that multiple DSB ends are observed at distinct genomic positions. Second, most DSB events in Pv11 were supported by a single unique molecular identifier (UMI; Fig S3B), suggesting limited redundancy and/or low library complexity at individual break sites. Technical differences in cross-linking efficiency, end labeling, ligation, UMI collapsing rules, mapping stringency (including the choice of haploid reference and MAPQ thresholds), or cell-number estimation could also affect the absolute DSB-per-cell estimate.

**Figure S4.**
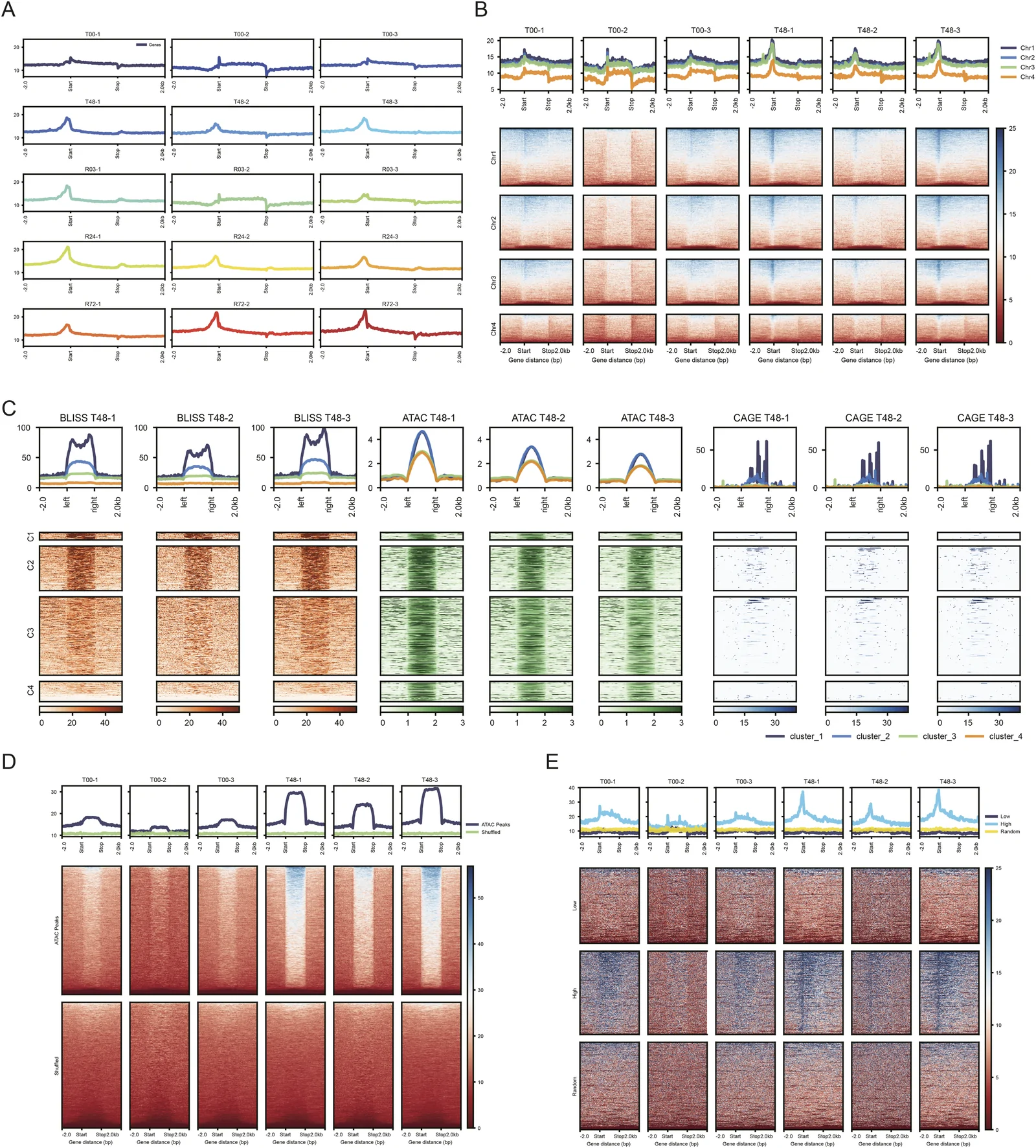
sBLISS profiling across the anhydrobiosis cycle. Pv11 cells were collected before (T00) and after (T48) the 48-h trehalose preconditioning. **(A)** Genome-wide DSB distributions across gene features for all conditions. DSB frequencies were aggregated along scaled gene models. Because untranslated regions were not annotated, “start” and “end” correspond to the start and stop codons, respectively. **(B)** Chromosome-level visualization of DSBs in trehalose-treated samples (T00 and T48). Enrichment near start codons is observed. **(C)** Integrated sBLISS, ATAC-seq, and CAGE-seq signals at ATAC-seq peaks for the three T48 replicates. Replicates show minimal variation. **(D)** DSB signal distributions within open chromatin regions defined by ATAC-seq peaks. **(E)** DSB profiles at ATAC-seq peaks overlapping TSSs of genes in the top and bottom 5% expression bins (CAGE-seq). Random genomic regions of matched length were used as controls.

As a control, sBLISS was also performed on Pv11 cells treated with 1 mM hydrogen peroxide, an inducer of oxidative DNA damage. Compared with the anhydrobiosis time course, promoter-proximal enrichment was not observed (Figs S3C and S4A). This difference may reflect distinct damage chemistry or differences in the spatial distribution of lesions under exogenous hydrogen peroxide treatment, which induces DNA damage primarily through oxidative stress and downstream radical formation.

Overall, these analyses indicate that absolute DSB-per-cell estimates in Pv11 should be interpreted cautiously because they are affected by genome size, library complexity, UMI recovery, mapping stringency, and cell-number estimation. In contrast, relative DSB enrichment patterns across genomic features were reproducible and informative. We therefore used sBLISS primarily to compare the genomic distribution of DSBs between conditions rather than to infer absolute numbers of breaks per cell.

Across datasets, 3–20 million mapped DSB events were detected (Tables S9 and S10). Because DSB counts did not decrease after rehydration (Fig S4A) and >80% of rehydrated cells were nonviable by Hoechst/propidium iodide staining, rehydration datasets were considered confounded by background from dead cells and were excluded from subsequent comparative analyses. We therefore focused subsequent analyses on the trehalose pretreatment samples (T00 and T48).

Table S9. sBLISS statistics. Number of DSBs detected in all sBLISS datasets generated in this study.

Table S10. sBLISS DSB locations. Enrichment analysis of DSB locations detected in each sBLISS dataset.

DSB localization was initially quantified relative to BRAKER-annotated gene structures. Promoter-proximal regions (<2 kb upstream of the start codon), exons, and transcription termination sites (<2 kb downstream of the stop codon) showed elevated DSB signal (Fig S4A and B), consistent with patterns reported in mammalian cell lines (Bouwman et al, 2020).

To test whether these enrichments were centered on transcription start sites (TSSs), we reanalyzed previously published CAGE-seq data (Yamada et al, 2018). The ReapTEC approach was used to identify TSSs. This method filters CAGE tags by exploiting a protocol-derived feature: an artificial guanine is introduced at the 5′ end during library preparation, and genuine capped transcript reads therefore tend to show a 5′ G signature (often observed as a soft-clipped 5′ G in alignments). Applying this filter reduces total read count available for clustering but substantially decreases background signal, as evidenced by fewer clustered TSS (cTSS) calls outside annotated promoter-proximal regions.

**Figure S5.**
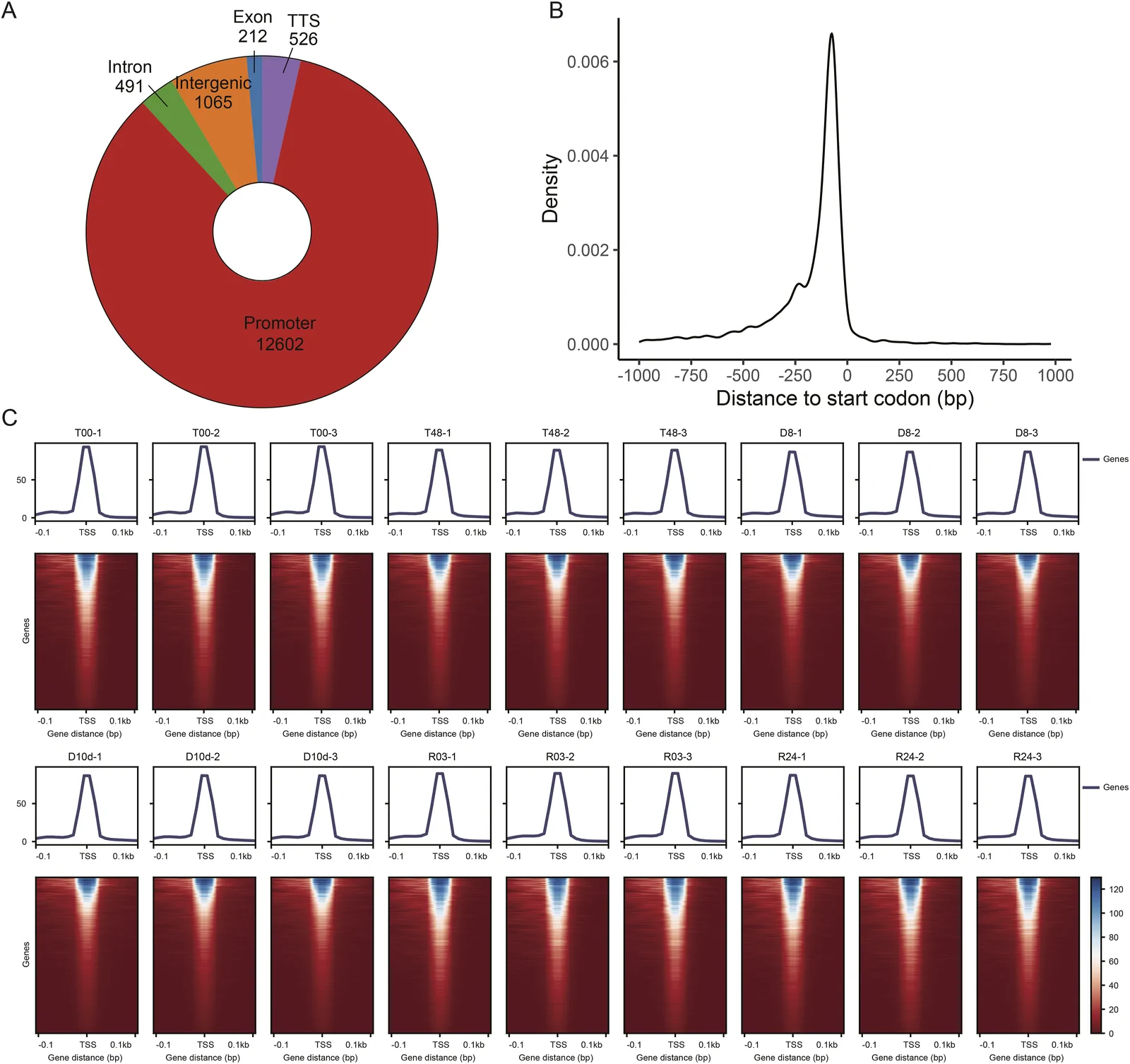
Identification of TSSs using CAGE-seq. **(A)** Genomic distribution of CAGE-defined cTSSs. Previously published CAGE-seq data (Yamada et al, 2018) were reprocessed to identify cTSSs, which were then annotated by position relative to gene features. **(B)** Distance from cTSSs to the nearest start codon on the same strand. Most cTSSs fall within 250 bp of the closest start codon. **(C)** Aggregate CAGE tag profiles around cTSSs. Coverage within ±100 bp of cTSS positions is shown. Pv11 cells were collected before (T00) and after (T48) the 48-h trehalose preconditioning, as well as 8 h (D8) and 10 d (D10d) after drying, and 3 h (R3) and 24 h (R24) after rehydration.

Using ReapTEC-filtered tags, 14,896 cTSSs were identified (Fig S5A), and 83.3% were located within 1 kb of an annotated start codon on the corresponding strand. Because BRAKER gene models are defined by CDS boundaries, proximity was measured to the start codon. Without ReapTEC filtering, 14,582 cTSSs were detected, of which 80.5% fell within the same distance threshold, indicating slightly reduced specificity. Approximately 7% of cTSSs were located in intergenic regions, potentially reflecting unannotated transcripts or noncoding RNAs. Most cTSSs were positioned within 250 bp of the nearest start codon on the same strand (Fig S5B), suggesting relatively short 5′ untranslated regions in Pv11. Coverage visualization confirmed a narrow peak centered at cTSS positions (Fig S5C). To characterize promoter-proximal sequence features, motif enrichment analysis was performed using HOMER on the 100 bp upstream of each cTSS. Several motifs were significantly enriched, including *Drosophila*-like initiator, E-box, and TATA-box motifs. Together, these analyses support the overall quality of cTSS annotation in Pv11 and provide a resource for future studies of transcriptional regulation. Concordantly, DSB tags accumulated within −100 to +100 bp of cTSSs (Fig 4B), and the resulting TSS-centered profiles showed a periodic pattern with ∼150- to 200-bp spacing, suggestive of nucleosome phasing.

**Figure S6.**
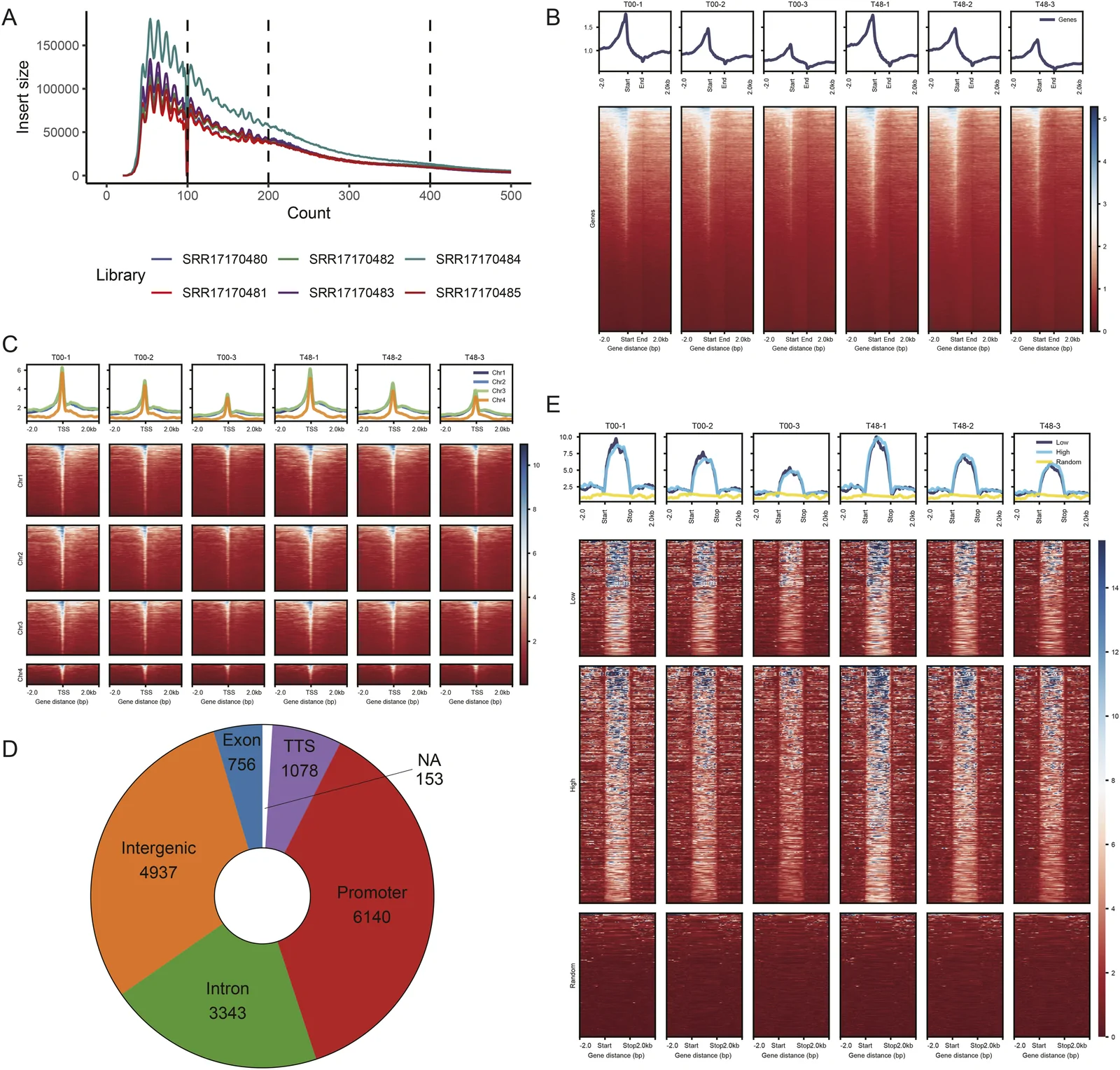
Detection of open chromatin regions using ATAC-seq. Pv11 cells were collected before (T00) and after (T48) the 48-h trehalose preconditioning. **(A)** Insert-size distribution of ATAC-seq fragments. Canonical periodic peaks indicative of high-quality libraries are weak or absent, indicating moderate library quality. Black dotted lines indicate 100, 200, and 400 bp. **(B, C)** ATAC-seq coverage across scaled gene bodies (start to stop codon) and around CAGE-defined cTSSs, respectively. ATAC-seq signal is enriched upstream of gene bodies and near TSSs. **(D)** Genomic distribution of ATAC-seq peaks. Peaks called by Genrich were annotated based on their positions relative to gene features. NA: not annotated. **(E)** ATAC-seq coverage at peaks associated with high and low expression genes (as defined in Fig S4E). Random genomic regions of matched length were used as controls.

This periodicity motivated the hypothesis that chromatin accessibility influences DSB susceptibility at promoters. We therefore used previously published ATAC-seq data to define open chromatin regions and quantified DSB levels around ATAC-seq peaks. As an initial quality check, the insert-size distribution was evaluated. High-quality ATAC-seq libraries typically show periodic peaks near ∼200, 400, and 600 bp, corresponding to mono-, di-, and trinucleosome fragments. In the present dataset, library complexity was high across all ATAC-seq libraries (NRF and PBC1 ∼1.0; PBC2 extremely high, consistent with minimal duplication), but nucleosome periodicity in the fragment-size distribution was weak (Fig S6A), indicating limited signal-to-noise for fine-scale regulatory inference. Approximately 55–60% of the alignments were assigned to Genrich peaks. Despite this limitation, downstream analyses were performed to enable integrated comparison with sBLISS and CAGE-seq signals.

ATAC-seq signal was enriched near gene starts (Fig S6B) and near CAGE-defined cTSSs (Fig S6C), consistent with accessibility at promoter-proximal regions. Peak calling with Genrich identified 16,407 ATAC-seq peaks (Fig S6D), of which 37% overlapped annotated TSSs. Approximately 20% of peaks fell within introns, which could reflect true intronic regulatory elements and/or false positives arising from library limitations. ATAC-seq peaks were intersected with cTSSs associated with the top and bottom 5% of gene expression. High ATAC-seq coverage was observed at peaks linked to both highly and lowly expressed genes (Fig S6E). This pattern suggests that chromatin accessibility alone does not fully explain steady-state expression in this dataset and that peak-to-gene assignment, temporal regulation, or data quality may contribute. More robust ATAC-seq data will be required for high-confidence genome-wide inference of regulatory architecture.

Peaks with higher accessibility (ATAC clusters 1 and 2) showed stronger sBLISS signal and higher CAGE activity (Figs 4C and S4C). The distributions of DSB signals within these ATAC-seq–defined open chromatin regions are shown in Fig S4D. Not all ATAC-seq peaks exhibited detectable DSB enrichment, which may partly reflect false-positive peaks arising from weak nucleosome periodicity in the ATAC-seq insert-size distribution (Fig S6A) and/or other library limitations. Consistent with an accessibility–transcription relationship, cTSSs associated with the top 5% of expressed genes exhibited higher DSB frequencies than those in the bottom 5% (Figs 4D and S4E). Together, these analyses indicate that open, transcriptionally active promoter-proximal regions are preferential sites of DSB accumulation in Pv11.

## Discussion

In this study, a haplotype-resolved diploid genome assembly was established for the Pv11 cell line and used to (i) characterize haplotype diversity, particularly on Chr4, (ii) evaluate chromosome-scale gene order evolution across chironomids, and (iii) map genome-wide DNA DSB landscapes during trehalose preconditioning.

### Establishment of a diploid genome for the Pv11 cell line

The v7.0 Pv11 assembly substantially improves upon the previous reference (Yoshida et al, 2022b), as supported by multiple quality metrics. Despite this progress, repetitive and structurally complex regions remain a primary constraint for a complete assembly. Consistent with this, the Pv11 genome retained canonical chromosome-end and centromere-associated features that are difficult to reconstruct from finite long-read coverage.

Two repeat unit candidates (150 and 303 bp) were identified at chromosome termini. The 303-bp unit appeared more completely repeated, whereas the 150-bp unit frequently contained truncated variants interspersed within full-length sequences. Candidate telomeric repeats reported in *Chironomus* (Saiga & Edstrom, 1985; Cohn & Edstrom, 1992) were not detected in Pv11, nor in available assemblies of the close relatives *Po. nubifer* (Gusev et al, 2014) and *Po. pembai* (Shaikhutdinov et al, 2023). Although this pattern is compatible with, but does not establish, rapid telomeric sequence turnover in *Polypedilum*, interpretation should remain cautious given assembly fragmentation in short-read–based references and the possibility that telomeres are underrepresented in some datasets.

Centromeres have been observed cytogenetically in *Po. vanderplanki* (Petrova et al, 2015), yet no definitive centromeric repeat units were identified here, mirroring limitations in other chironomid genomes. Centromeres and telomeres are notoriously hard to assemble correctly, illustrated by the human T2T assembly (Nurk et al, 2022). Previously proposed centromeric candidates from *Chironomus* (Rovira et al, 1993) were also absent from the Pv11 cell line. Together, the apparent divergence of telomeric and centromeric sequences, even among related chironomid lineages, highlights a tractable system to study chromosome-end evolution and genome stability once additional high-quality references become available. CENP-A/CenH3 ChIP-seq and cytogenetic mapping would provide information on the location and structures of centromeres.

Several gaps persisted despite incorporating Nanopore long reads into the hifiasm assembly. A likely contributor is the limited effective ultra-long coverage from ONT R9 chemistry; after filtering for reads >50 kb, coverage was ∼10×, which is typically insufficient to traverse the most complex regions. In addition, the available Nanopore data were generated from Pv11-KH, a derivative cell line of Pv11 (Miyata et al, 2022), which carries a randomly integrated AcGFP1 expression cassette. No AcGFP1 sequence was detected in v7.0, consistent with minimal impact of KH-derived reads on the final consensus, although residual effects cannot be fully excluded without matched ultra-long data from Pv11 SCI-B.

A gapless telomere-to-telomere assembly will likely require additional ultra-long ONT sequencing. Such data would help resolve several open questions raised by the current assembly. One possibility is that Chr2 and Chr3 are fused, as suggested by genome-graph connectivity and by the absence of telomeric repeats at the relevant chromosome ends. The assembly graph connected Chr2 and Chr3, but neither the genome-wide Hi-C map nor the available long-read alignments resolved an unambiguous junction. Although reads mapped to the regions flanking both scaffold ends, no read provided a definitive bridge between them. The absence of detectable Unit_A or Unit_B sequences at these scaffold ends could also reflect incomplete assembly of terminal repeats. Thus, the current data neither confirm nor exclude a Chr2-Chr3 fusion. Direct cytogenetic validation would be informative; however, robust cell-cycle synchronization methods for Pv11 cells remain a major practical barrier. Second, the cause of the extremely low heterozygosity observed across portions of Chr2 and Chr3 remains unclear and may reflect lineage history, selection, or structural constraints (Fig 2). Third, incomplete reconstruction of telomeric regions limits analyses of telomere architecture and its dynamics under genotoxic stress.

Overall, the v7.0 diploid assembly provides a robust foundation for evolutionary and genetic analyses that were previously compromised by haplotype switching and local misassembly. Despite its high base accuracy and chromosome-scale organization, the v7.0 assembly should be considered a high-quality chromosome-level haplotype-resolved assembly rather than a complete or telomere-to-telomere genome, because several chromosome-scale scaffolds retain gaps and a substantial number of contigs remain unplaced. Beyond comparative genomics, the Pv11 platform is increasingly positioned for cell engineering, including recently developed biosensor applications (Fuse et al, 2025). The availability of a diploid reference should enable more accurate genome editing, including haplotype-aware targeting and other precision manipulations.

### Use of the diploid genome for evolutionary analysis

Improved assembly accuracy enables more reliable inference of gene conservation, divergence, and structural variation than was possible with earlier references. A central advantage of a diploid assembly is the ability to quantify haplotype differences directly, including SNVs and structural variation, and to relate these differences to gene content and expression.

Consistent with earlier work, Chr4 again showed pronounced divergence between haplotypes, with SNVs enriched around genes and a measurable fraction of genes present on only one haplotype. Notably, ∼20–25% of haplotype-specific genes localized to Chr4, reinforcing the view that this chromosome is unusually dynamic. These observations initially suggested that Chr4 may experience relaxed selective constraint. However, analyses across independently cultured Pv11 populations did not reveal a Chr4-specific increase in nucleotide diversity, arguing against ongoing, Chr4-restricted drift during cell culture. A parsimonious interpretation is that elevated Chr4 heterozygosity largely predates the separation of the sampled Pv11 populations and may partly reflect variation already present at or before cell-line establishment. At present, however, the available data do not distinguish among increased mutation input, altered selective constraint, and demographic effects as alternative explanations for Chr4 divergence.

The diploid reference also strengthens comparative analyses of chromosome evolution in chironomids. Cytogenetic studies of *Chironomus* have documented extensive inversion polymorphism and structural chromosome variation (Kiknadze, 2008), and some chromosomal breakpoints colocalize with transposable elements or tandemly repetitive DNA (Bovero et al, 2002; Michailova et al, 2009). To examine chromosome evolution across different phylogenetic depths within the family Chironomidae, we compared gene-level synteny among Pv11 cells, derived from *Po. vanderplanki*, *C. riparius*, and *Pr. akamusi*. *Po. vanderplanki* and *C. riparius* belong to the subfamily Chironominae, whereas *Pr. akamusi* is treated here as a member of Orthocladiinae sensu lato, although it has also been classified within Prodiamesinae (Semenchenko et al, 2025; Xu et al, 2026).

All three pairwise comparisons showed highly fragmented synteny blocks. In the comparison between Pv11 and *C. riparius*, gene order was extensively reshuffled within chromosomes, whereas broad chromosome identity was retained. Comparisons involving the more distantly related *Pr. akamusi* showed similarly fragmented synteny, indicating that this pattern is not restricted to a single species pair or to divergence within Chironominae.

Nevertheless, this three-species comparison does not establish that extensive intrachromosomal reorganization is universal across Chironomidae, nor does it resolve the ancestral chromosome arrangement or the direction and rate of individual rearrangements. Differences in sequence divergence, assembly quality, gene annotation, and ortholog detection may also contribute to the apparent fragmentation of synteny blocks. Additional chromosome-level genomes representing other chironomid subfamilies will therefore be required to determine how broadly this pattern occurs across the family.

Within the Pv11-to-*C. riparius* comparison, elevated Ka/Ks values were observed among Chr4 genes with minimal expression, raising the possibility of relaxed constraint in transcriptionally inactive loci. However, the magnitude of Ka/Ks for minimally expressed Pv11–*C. riparius* orthologs (mean ∼0.1) remained far below values typically associated with pseudogenization and was also markedly lower than the average dN/dS (∼0.55) previously observed for a highly duplicated ionotropic receptor family in *Po. vanderplanki*. These comparisons support continued purifying selection on conserved orthologs, with the strongest relaxation confined to lowly expressed genes rather than widespread functional decay.

### Understanding the genome-wide DNA damage landscape

Genome integrity has been a recurring theme in studies of anhydrobiotic animals (Gladyshev & Meselson, 2008; Rebecchi et al, 2009; Gusev et al, 2010; Anoud et al, 2024; Li et al, 2024). The frequent observation of radiation resistance has motivated the hypothesis that these organisms possess efficient DNA repair capacity. Most prior work has emphasized survival phenotypes and DNA damage measurements in irradiated individuals or after rehydration, whereas direct analyses of transcriptional programs during repair remain comparatively limited (Yoshida et al, 2021, 2022a, 2024). In *Po. vanderplanki* and Pv11, transcriptional induction of key repair pathways, including homologous recombination and nonhomologous end joining, has been reported (Gusev et al, 2014; Yamada et al, 2018). Nevertheless, persistent DNA damage up to 72 h after rehydration in larvae suggests that genome protection during desiccation can be incomplete (Gusev et al, 2010). This motivated the use of the homogeneous Pv11 cell system to resolve genome-wide DSB landscapes under controlled conditions, without the confounding tissue heterogeneity present in whole larvae.

sBLISS detected DSB events across all sampled stages of the anhydrobiosis cycle. Downstream interpretation focused on trehalose pretreatment because rehydration samples contained a high fraction of dead cells. DSB-associated signals can persist in nonviable material and inflate apparent break counts across time points, obscuring recovery dynamics. Future work incorporating dead-cell depletion or viability sorting should enable more definitive mapping of repair-associated changes during rehydration.

Although DSB enrichment during desiccation or rehydration was initially expected, substantial DSB signals were also observed during trehalose pretreatment. Trehalose exposure generates a strong hyperosmotic environment (600 mM) that can drive cell shrinkage and mechanical stress. Hyperosmolarity-induced DNA damage remains incompletely characterized in mammalian systems (Zhang & Fan, 2025), and the Pv11 data raise the possibility that hyperosmotic stress contributes to DSB formation in this model.

DSBs were enriched near transcription start regions of highly expressed genes, which coincide with open chromatin. This pattern parallels observations in mammalian cells (Bouwman et al, 2020) and is consistent with the idea that accessible, transcriptionally engaged loci are more vulnerable to DNA damage. During the 48-h trehalose pretreatment, anhydrobiosis-associated genes are strongly induced to prepare for subsequent stress and recovery (Yamada et al, 2018). High transcriptional activity at these loci may increase damage susceptibility, whereas their essential roles likely impose strong purifying selection that limits long-term accumulation of deleterious variants.

In contrast, hydrogen peroxide treatment did not recapitulate the same enrichment pattern near promoter-proximal features. One explanation is that the predominant reactive oxygen species and damage chemistry differ between hyperosmotic preconditioning and exogenous H_2_O_2_ exposure. Hydrogen peroxide can generate highly reactive hydroxyl radicals through transition metal–catalyzed Fenton reactions, thereby inducing various forms of DNA damage, including DSBs (Prise et al, 1989). The major endogenous ROS during anhydrobiosis remains unclear, but genome-based analyses have reported expansion of thioredoxin family genes in an anhydrobiosis-related gene cluster island (ARId5), and induction of glutathione S-transferases and superoxide dismutases has been observed, consistent with multiple ROS types during drying (Gusev et al, 2014; Nesmelov et al, 2018). In addition, the relatively high H_2_O_2_ concentration used here (1 mM) may have produced widespread damage that masks locus-specific susceptibility patterns apparent under more physiological stress intensities.

A second, nonexclusive possibility is that trehalose pretreatment engages stress-response and DNA damage–response programs that may influence the observed break distribution. Paraquat exposure induces anhydrobiosis-related genes in *Po. vanderplanki* larvae (Cornette et al, 2023), suggesting cross-talk between superoxide stress responses and the desiccation-response cascade. High-salt exposure, which also induces hyperosmolarity (Tamura et al, 2013), triggers trehalose accumulation in larvae (Watanabe et al, 2003), further linking osmotic stress to the anhydrobiosis program. Cross-talk between desiccation tolerance and radiation responses has been reported in *Po. vanderplanki* (Ryabova et al, 2017) and in tardigrades (Yoshida et al, 2021, 2022a, 2024; Li et al, 2024).

Collectively, our data show that trehalose pretreatment is accompanied by a nonrandom DSB landscape, with breaks preferentially accumulating near accessible highly expressed TSSs, which was not observed under the H_2_O_2_ exposure. These findings support a working model in which promoter accessibility and transcriptional activity influence local DSB susceptibility during trehalose-induced preparation for anhydrobiosis. However, the present study does not establish causal relationships among hyperosmotic stress, chromatin state, transcription, DSB formation, and DNA repair, which will require direct functional testing.

## Materials and Methods

### Cell culture and establishment of a clonal line

Pv11 cells were cultured as previously described (Nakahara et al, 2010). Cells were maintained at 25°C in complete IPL-41 medium consisting of IPL-41 Insect Medium (Thermo Fisher Scientific) supplemented with 2.6 g/liter tryptose phosphate broth (BD), 10% (vol/vol) FBS (HyClone, Cytiva), and 0.05% (vol/vol) antibiotic–antimycotic (100×) solution (Thermo Fisher Scientific). Cultures were passaged weekly by gentle pipetting and diluted to 1.5 × 10^5^ cells/ml in fresh complete medium. A clonal cell line, Pv11 SCI-B, was established by two rounds of limiting dilution.

To induce desiccation tolerance, Pv11 cells were incubated in a preconditioning medium (600 mM trehalose solution containing 10% (vol/vol) complete IPL-41 medium) for 48 h at 25°C. Cells were then resuspended in 400 μl of the preconditioning medium, and 40 μl aliquots were dropped into 35-mm petri dishes. The dishes were placed in a desiccator maintained at <10% relative humidity and kept at 25°C for more than 10 d. The desiccated cells were rehydrated with complete IPL-41 medium and incubated at 25°C.

### Assembly of the Pv11 genome (v6.0) and chromosome-scale scaffolding (v6.1)

PacBio CLR reads from our previous study (NCBI accession ID: SRR12736666) were assembled with NextDenovo v2.5.0 (Hu et al, 2024). The assembly was polished with NextPolish v1.4.0 (Hu et al, 2020) using both CLR reads and Illumina short reads from samples idx05 (NCBI accession ID: SRR12736661) and idx19 (NCBI accession ID: SRR12736660) (Yoshida et al, 2022b). A mitochondrial contig was identified and removed by minimap2 search against the reference mitogenome (NCBI accession ID: KT251040) (Deviatiiarov et al, 2017), yielding the Pv11_6.0 assembly.

For chromosome-scale scaffolding, Hi-C reads from our previous study (Yoshida et al, 2022b) (NCBI accession ID: SRR12736658, SRR12736657) were processed using the Arima Genomics mapping pipeline (https://github.com/ArimaGenomics/mapping_pipeline). Briefly, reads were aligned to the assembly with BWA-MEM2 v2.2.1 (Vasimuddin et al, 2019) and processed with SAMtools v1.7 (Li et al, 2009). PCR duplicates were marked using Picard v2.26.11 (Broad Institute, 2026). Reads were further filtered using a custom Perl script, and scaffolding was performed with YaHS v1.2a.2 (Zhou et al, 2023) together with the Juicer pipeline v1.1 (Durand et al, 2016). Scaffolding quality was evaluated using Juicer Tools v1.22.01 and visualized in Juicebox v3.01.04 (Dudchenko et al, 2018 *Preprint*), generating the Pv11_6.1 assembly.

For gene prediction on Pv11_6.0, repeats were masked using a custom *Po. vanderplanki* repeat library and RepeatMasker v4.1.2-p1 (http://www.repeatmasker.org) (Smit et al, 2013–2015). RNA-seq data (NCBI GEO accession ID: GSE158443) from Yamada et al (2020) and Yoshida et al (2022b) were aligned with HISAT2 v2.2.1 (Kim et al, 2019) and processed with SAMtools v1.7. Gene models were generated using BRAKER1 and BRAKER2 v2.1.6 (Hoff et al, 2016; Bruna et al, 2021) with evidence from RNA-seq and the OrthoDB Arthropoda protein dataset (downloaded March 15, 2022) (Kriventseva et al, 2015). BRAKER1 models that did not overlap BRAKER2 predictions were merged into the BRAKER2 gene set using BEDTools v2.30.0 (Quinlan & Hall, 2010). Well-characterized anhydrobiosis-related genes were curated manually.

### Hi-C–based phasing and diploid reassembly (v6.2)

To construct a phased assembly from Pv11_6.1, variants previously identified from idx05 and idx19 gDNA-seq libraries using BCFtools v1.8 (Li, 2011) were phased with HapCut2 (Edge et al, 2017) within a Hi-C Snakemake workflow (bwatools v2.5.1, BWA v0.7.17, HapCut2 v1.1, SAMtools v1.14, Picard v2.26.10). Phased variants were merged with BCFtools v1.8, and PacBio reads were assigned to haplotypes using WhatsHap v1.7 (Martin et al, 2016 *Preprint*). Reads were split into haplotype-specific sets (hp1 + hp0 and hp2 + hp0; hp0 denotes unphased reads) using SAMtools v1.15 and reassembled with NextDenovo v2.5.0.

Assembly completeness was assessed with compleasm v0.2.5 using the Diptera lineage dataset (Huang & Li, 2023). Consensus quality was evaluated with Merqury v1.3 (Rhie et al, 2020) using a hybrid k-mer database constructed as described below. Gene models were transferred to Pv11_6.1 using Liftoff v1.6.3 (Shumate & Salzberg, 2021).

### Genome sequencing and assembly (v7.0)

High-molecular-weight genomic DNA was extracted from ∼10^7^ Pv11 SCI-B using MagAttract HMW DNA Kit (QIAGEN). Purified DNA (10 μg) was submitted to NIG Japan for PacBio HiFi sequencing through the PAGS support program. Sequencing on a PacBio Sequel IIe generated 14.8 Gb of HiFi data, corresponding to ∼123.2× coverage of the haploid genome.

In addition, short-read sequencing was performed for a recent Pv11 population before clonal line derivation. Genomic DNA was extracted using NucleoBond HMW DNA Kit (TaKaRa Bio) and sequenced by Bioengineering Lab Co., Ltd. DNA was fragmented to ∼500 bp using a Covaris system, and libraries were prepared using MGIEasy PCR-Free DNA Library Prep Set. After circularization with MGIEasy Circularization Kit, sequencing was carried out on a DNBSEQ-G400RS instrument using High-Throughput Sequencing Kit. This dataset was designated Pv11_S27.

HiFi reads were assembled with hifiasm v0.19.9-r616 (Cheng et al, 2021, 2022). Previously published Hi-C data (NCBI accession ID: SRR12736658, SRR12736657) (Yoshida et al, 2022b) were incorporated, together with ONT long reads from a GFP-inserted Pv11 derivative (Miyata et al, 2022) (NCBI GEO accession ID: GSE190481; GSM5724412 and GSM5724413; 2.764 Gb total; ∼10× diploid genome coverage; max read length 310,787 bp; read N50 70.54 kb; SRA: SRR17170486 and SRR17170487). Only ONT reads longer than 50 kb were used (13.27% of the reads were retained).

The phased diploid assembly was scaffolded using the allele-aware HapHiC tool v1.0.3 (Zeng et al, 2024). Hi-C reads were aligned to the assembly with BWA-MEM and filtered using SAMBLASTER v0.1.26 (Faust & Hall, 2014). The resulting BAM files and diploid GFA were processed with the HapHiC pipeline to generate chromosome-level scaffolds. Scaffolds were validated and oriented using Juicer/Juicebox and compared with the Pv11_5.2 assembly. Gap filling was attempted using TGS-GapCloser v1.2.1 (Xu et al, 2020) but did not produce satisfactory improvements. The final hifiasm assembly graph was annotated by aligning contigs to Pv11_5.2 with minimap2 and visualized using Bandage.

### Error correction and final assembly evaluation

The assembly was polished using the T2T-Polish pipeline (https://github.com/arangrhie/T2T-Polish) (Mc Cartney et al, 2022). To evaluate initial consensus quality, an 18-mer hybrid meryl database v1.3 was constructed from k-mers derived from both Pv11_S27 short reads and PacBio HiFi reads. K-mers with counts >1 were retained, and the union-sum was used to build a hybrid meryl database, which was provided to Merqury for quality evaluation (Rhie et al, 2020).

For polishing, 15-mers were counted from raw HiFi reads using meryl, and highly repetitive k-mers (distinctness > 0.9998) were flagged. HiFi reads were mapped using Winnowmap v2.03 (--MD -ax map-pb) with the repetitive 15-mers excluded. Primary alignments were retained using SAMtools v1.20 (view -F 0x104). Reads were further filtered by the presence of 18-mers meeting the following criteria: (i) unique in the assembly, and (ii) present ≥50 and <125 times in the hybrid meryl database (k18_gt1). These filters enriched for reads originating from single-copy regions with sufficient depth.

Structural variants (SVs) and small variants were then identified and corrected. SVs were called from HiFi alignments using Sniffles v2.3.3 (Sedlazeck et al, 2018), refined with Iris v1.0.5 (Kirsche et al, 2023), and consolidated with Jasmine v1.1.4 (Kirsche et al, 2023). Small variants were called using DeepVariant v1.6.0 (--haploid_contigs) (Poplin et al, 2018) and filtered with BCFtools v1.11 (-f “PASS” -e “FORMAT/VAF<=0.5 | FORMAT/GQ<=30”). Variants were applied using Merfin v1.10 (-polish -peak 60) (Formenti et al, 2022) with the k18_gt1 meryl database. SV and small-variant calls were merged and manually inspected in JBrowse2 (Diesh et al, 2023), and validated corrections were applied using BCFtools consensus.

Residual anomalies were assessed using issues.sh from the T2T-Polish pipeline (commit 2e81f6d5fe5684e6f78932046687552db042ec06). Assembly quality was further evaluated using Merqury (hybrid meryl database), compleasm (Diptera lineage), SeqKit stats (Shen et al, 2016), and standard assembly statistics. To remove mitochondrial contamination, the *Po. vanderplanki* mitochondrial genome (NCBI accession ID: KT251040.1) was aligned to the final assembly with minimap2 v2.28-r1209, and six scaffolds corresponding to mitochondrial sequences were excluded.

### Genomic feature annotation

Repeat families were characterized using RepeatModeler v2.0.5 (Flynn et al, 2020), followed by genome masking with RepeatMasker v4.1.5. For gene prediction, previously published RNA-seq datasets (NCBI GEO accession ID: GSE158443) (Yamada et al, 2020; Yoshida et al, 2022b) were mapped to the genome with HISAT2 v2.2.1 (--very-sensitive). RNA-seq alignments and the OrthoDB Arthropoda (downloaded January 29, 2024) protein dataset were used as inputs for BRAKER3 v3.0.8 (Gabriel et al, 2024). Because a BRAKER3 pipeline error, many predicted models contained duplicated features differing only at the stop-codon region; these redundancies were removed using a custom script.

Protein-coding sequences and amino acid translations were generated using gffread v0.12.7 (Pertea & Pertea, 2020). Functional annotation was performed with InterProScan v5.68-100.0 (Jones et al, 2014). Horizontally transferred genes were identified using AvP v1.0.9 (Koutsovoulos et al, 2022). Candidate telomeric repeat units were first identified by manual inspection of chromosome termini and were then used as custom libraries in RepeatMasker v4.1.5 to assess their genomic distribution. Candidate centromeric sequences were screened with CentroAnno (Qi et al, 2025 *Preprint*), without any motif templates.

To characterize the sequence feature underlying the Chr4 gap (Hap1: 2,496,276; Hap2: 2,159,598), we manually inspected the corresponding genomic interval in JBrowse2 and extracted the relevant sequence using SeqKit (grep and subseq). Tandem Repeats Finder v4.09.1 was used to identify repeat consensus sequences with the following parameters: 2 5 7 80 10 50 2000 -l 10 -h.

### Variant detection between haplotypes

#### Direct haplotype-to-haplotype comparison

Hap1 chromosomes were aligned to their corresponding Hap2 chromosomes using minimap2 v2.28-r1209 (-ax asm20 --eqx). Variants were identified using SyRI v1.7.0 (-F S -k --cigar --hdrseq) (Goel et al, 2019). Per-chromosome callsets were concatenated, and SNVs were aggregated into 10-kb windows using BEDTools intersect v2.30.0. Variants were annotated using SnpEff v5.2 (ann -ud 2000) (Cingolani et al, 2012) to classify positions relative to gene features.

#### Read-based variant calling against the alternate haplotype

HiFi reads were mapped to the diploid assembly using Winnowmap v2.03 with repetitive 15-mers excluded. Primary alignments were retained (SAMtools v1.20 -F 0x104). Reads were extracted by primary mapping chromosome and remapped to the corresponding chromosome of the alternate haplotype (e.g., Chr1_Hap1-mapped reads were remapped to Chr1_Hap2) using Winnowmap. Variants were called with DeepVariant v1.6.0 (--model_type PACBIO--haploid_contigs=<chromosome>) and filtered with BCFtools v1.20 (-f “PASS” -e “FORMAT/VAF<=0.5 | FORMAT/GQ<=30”). Filtered calls were merged to generate the final callset. SNVs were summarized in 100-kb windows using BEDTools.

### Population-level variant analysis using short reads

Short-read datasets from previous studies (NCBI SRA accession IDs: SRR12736659, SRR12736660, SRR12736661, SRR12736662, DRR024749) and the new Pv11_S27 dataset were analyzed to quantify variation across Pv11 populations. Reads were trimmed with Trimmomatic v0.39 (LEADING:30 TRAILING:30 SLIDINGWINDOW:4:15 MINLEN:30) (Bolger et al, 2014) and aligned to the diploid assembly with BWA-MEM2 v2.2.1 (Vasimuddin et al, 2019). PCR duplicates were marked using Picard MarkDuplicates v3.3.0 (Broad Institute, 2026), and primary alignments were retained (SAMtools -F 0x104).

Initial SNV calling was performed with GATK Mutect2 v4.6.1.0 (Van der Auwera & O’Connor, 2020). Read orientation bias was modeled with LearnReadOrientationModel, and variants were filtered with FilterMutectCalls. Base quality score recalibration was performed using BaseRecalibrator and ApplyBQSR, followed by a second Mutect2 run. Filtered SNVs were extracted using BEDTools (-f PASS -v snps) and annotated with SnpEff.

CNV analysis was performed using GATK CollectReadCounts (--interval-merging-rule OVERLAPPING_ONLY) and CollectAllelicCounts. Read-depth denoising was performed using DenoiseReadCounts with HiFi data as a panel of normals, and CNVs were inferred using ModelSegments and CallCopyRatioSegments. Average coverage was calculated in 1,000-bp bins using BEDTools, and regions exceeding 1.5× coverage were flagged using an in-house R script. After merging sample-specific results, nucleotide diversity (π) in 1,000-bp windows was calculated with VCFtools v0.1.16 (--window-pi 1000) (Danecek et al, 2011).

### Gene synteny analysis

Gene-level synteny among Pv11 (this study), *C. riparius*, and *Pr. akamusi* was analyzed using JCVI MCScan (Python version) v1.5.10 (Tang et al, 2024). The *C. riparius* genome was obtained from the Pest Genomics Initiative (https://www.pestgenomics.org/species/chironomus-riparius) (Pettrich et al, 2025). The *Pr. akamusi* genome and gene features (Sun et al, 2021) were obtained from a previous study (Nell et al, 2024). Primary transcripts were extracted from GFF files for all three species.

Orthologs and synteny blocks were identified using the compara.catalog and compara.synteny modules, and karyotype visualizations were generated using graphics.karyotype. Selective constraint on synteny-defined ortholog pairs between Pv11 and *C. riparius* was evaluated by estimating Ka/Ks using anchor genes in compara.ks, calculated as Ka/Ks = yn_ka/yn_ks. LAST alignments produced during compara.catalog were also used to identify bidirectional best hits, which were used to quantify ortholog distributions across chromosome combinations.

### CAGE-seq data processing and analysis

CAGE-seq data were obtained from a previous study (NCBI BioProject PRJDB7516; NCBI SRA accession ID: DRR153208–DRR153225) (Yamada et al, 2018). Raw reads were filtered for rRNA contamination by mapping to the *Po. vanderplanki* rRNA locus using Bowtie2 v2.5.4 (Langmead & Salzberg, 2012). Remaining reads were mapped to the diploid assembly using both Bowtie2 v2.5.4 and HISAT2 v2.2.1. Because the CAGE protocol introduces an artificial guanine at the 5′ end of transcripts, this feature was used to filter reads that did not correspond to bona fide TSS following the cap-signature filtering principle used in ReapTEC (Oguchi et al, 2024). Reads lacking the 5′ G signature were removed.

Filtered CAGE tags were clustered using PROMOTERPIPELINE v20150516 (http://genome.gsc.riken.jp/plessy-20150516/PromoterPipeline_20150516.tar.gz). Clusters were categorized as “promoters” or “enhancers” using ChIPseeker v1.38.0 (Yu et al, 2015) based on distance (≤50 kb) to the nearest annotated gene. Cluster summits were defined as TSSs and annotated relative to predicted gene models using HOMER annotatePeaks.pl v4.11 (Heinz et al, 2010). TSSs in the top and bottom 5% of expression were identified using custom R scripts. Final BAM files were normalized using deepTools v3.5.5 bamCoverage with --effectiveGenomeSize 240387248 --binSize 10 --normalizeUsing RPGC --smoothLength 30 --Offset 1. Coverage matrices were generated using computeMatrix (--missingDataAsZero), and profiles and heatmaps were generated using plotProfile and plotHeatmap (Ramirez et al, 2016).

### sBLISS library preparation and sequencing

sBLISS libraries were prepared according to Bouwman et al (2020), with the Pv11-specific modifications described below. Protein LoBind and DNA LoBind tubes (Eppendorf) were used where appropriate to minimize sample loss.

#### Cell harvesting, hydrogen peroxide treatment, and fixation

Pv11 cell suspensions were gently pipetted several times and counted. For each condition, 5–10 × 10^6^ cells were collected in a 15-ml conical tube and centrifuged at 500*g* for 5 min at room temperature using a swing-bucket rotor. For samples collected immediately after the 48-h trehalose pretreatment, cells were washed twice with a 9:1 (vol/vol) mixture of preconditioning medium containing 600 mM trehalose (Fujifilm Wako) and DPBS+ (containing Ca^2+^ and Mg^2+^), with centrifugation at 500*g* for 5 min at room temperature after each wash.

For hydrogen peroxide controls, cells were washed with DPBS+, transferred to 1.5-ml conical tubes, and treated with 1 mM H_2_O_2_ for 20 min at room temperature with gentle rotation. Matched untreated samples were maintained in DPBS + for the same period. Cells were then centrifuged at 500*g* for 10 min at room temperature.

Collected cells were resuspended in 870 μl DPBS+, and then 130 μl methanol-free 16% PFA (Electron Microscopy Sciences) was added to give a final concentration of ∼2%. Cells were fixed for 10 min at room temperature with gentle rotation. Fixation was quenched by adding 2.5 M glycine to a final concentration of 125 mM, followed by incubation for 5 min at room temperature with gentle rotation and 5 min on ice. Fixed cells were centrifuged at 500*g* for 10 min at 4°C and washed twice with ice-cold DPBS without Ca^2+^ or Mg^2+^ (DPBS−), with centrifugation at 500*g* for 10 min at 4°C after each wash. The suspension was transferred to a new 15-ml tube during the second wash. Fixed cells were counted, resuspended in DPBS−, and stored at 4°C for up to 3 wk when not processed immediately. One million fixed cells were used for each sBLISS reaction.

#### Preparation of sBLISS SB adaptors

Upper and lower oligonucleotides for sBLISS SB adaptors were resuspended in TE buffer to 100 μM. For each SB, the lower oligonucleotide was phosphorylated in a 90-μl reaction containing 58 μl nuclease-free water, 10 μl 10× T4 polynucleotide kinase reaction buffer, 10 μl lower oligonucleotide (100 μM), 10 μl ATP (10 mM), and 2 μl T4 polynucleotide kinase (10 U/μl; NEB). Reactions were incubated at 37°C for 1 h, followed by heat inactivation at 65°C for 20 min. Ten microliters of the corresponding upper oligonucleotide (100 μM) was then added. Complementary strands were annealed at 95°C for 5 min and gradually cooled to 25°C. The resulting double-stranded adaptors were 10 μM and were stored at −20°C.

#### Cell lysis

One million fixed cells were transferred to a prechilled 1.5-ml Protein LoBind tube, centrifuged at 500*g* for 10 min at 4°C, and washed once with cold DPBS−. The pellet was resuspended in 200 µl LB1 buffer (10 mM Tris–HCl, pH 8.0, 10 mM NaCl, 1 mM EDTA, and 0.2% Triton X-100) and incubated on ice for 60 min, with gentle resuspension after 30 min.

Samples were centrifuged at 500*g* for 10 min at room temperature. Pellets were resuspended in 200 μl prewarmed LB2 buffer (10 mM Tris–HCl, pH 8.0, 150 mM NaCl, and 0.3% SDS) and incubated at 37°C for 60 min with gentle shaking. Samples were centrifuged at 500*g* for 10 min at room temperature and washed twice with 200 μl prewarmed CSTX buffer comprising 1× rCutSmart buffer (NEB) and 0.1% Triton X-100.

#### DSB blunting and adaptor ligation

For each sample, a 100-μl blunting reaction was prepared using Quick Blunting Kit (NEB): 75 μl nuclease-free water, 10 μl 10× blunting buffer, 1 μl 10 mg/ml BSA, 10 μl 1 mM dNTP mix, and 4 μl Blunt Enzyme Mix. Pellets were resuspended in the blunting mixture and incubated for 60 min at room temperature, with gentle resuspension after 30 min. Samples were centrifuged at 500*g* for 10 min at room temperature, washed twice with 200 μl CSTX buffer, and centrifuged at 500*g* for 5 min.

Four microliters of the selected 10 μM annealed sBLISS adaptor was added directly to each pellet, followed by 96 μl ligation mixture containing 76.8 μl nuclease-free water, 10 μl 10× T4 DNA ligase buffer, 1.2 μl ATP (100 mM), 3 μl BSA (50 mg/ml), and 5 μl T4 DNA ligase (5 Weiss U/μl; Thermo Fisher Scientific). The final 100-μl ligation reaction therefore contained 1.2 mM ATP, 1.5 mg/ml BSA, and 0.25 Weiss U/μl T4 DNA ligase. Samples were incubated at 16°C for 18–24 h.

#### Proteinase K treatment and DNA extraction

After ligation, samples were centrifuged at 500*g* for 10 min at room temperature and washed twice with 200 μl CSTX buffer. Pellets were resuspended in 100 μl Tail buffer (10 mM Tris–HCl, pH 7.5, 100 mM NaCl, 10 mM EDTA, and 0.5% SDS), supplemented with 10 μl proteinase K (800 U/ml; NEB), and incubated overnight at 55°C with shaking at 800 rpm at 3 mm swing width using a constant temperature incubator shaker (DWMax MBR-034; TAITEC). The next morning, samples that appeared clear received an additional 10 μl proteinase K and were incubated for 1 h at 55°C with shaking at 800 rpm using the constant temperature incubator shaker. Samples containing visible clumps received 100 μl Tail buffer and 30 μl proteinase K and were incubated for an additional 2 h under the same conditions. Proteinase K was heat-inactivated at 95°C for 10 min, and samples were cooled at room temperature for 10 min.

Sample volumes were adjusted with Tail buffer, and an equal volume of phenol:chloroform:isoamyl alcohol (25:24:1) was added. Samples were shaken vigorously for 30 s and centrifuged at 20,000*g* for 15 min at room temperature. The aqueous phase was transferred to a DNA LoBind tube, mixed with an equal volume of chloroform, shaken for 30 s, and centrifuged again at 20,000*g* for 15 min at room temperature.

DNA was precipitated by adding 0.1 volume of 3 M sodium acetate, glycogen (5 μl for ∼120 μl aqueous phase or 10 μl for ∼240 μl), and 2.45 volumes of ice-cold 100% ethanol. Samples were frozen at −80°C overnight and centrifuged at 30,000*g* for 90 min at 4°C. When no pellet was visible, 20–50 μl of 3 M sodium acetate was added and precipitation was repeated. Pellets were washed twice with ice-cold 70% ethanol, with centrifugation at 30,000*g* for 15 min at 4°C after each wash, air-dried for 10–15 min, and dissolved in 102 µl TE buffer at 50°C for 15 min with shaking at 1,100 rpm using the constant temperature incubator shaker.

#### Template shearing and cleanup

Extracted DNA (100 μl) was transferred to a 0.5-ml DNA LoBind tube and sonicated using a Bioruptor UCD-200T ultrasonic disruptor (Sonic Bio Co., Ltd.) for 10 cycles of 30 s ON and 90 s OFF. Sheared DNA was purified with 0.8× AMPure XP beads (Beckman Coulter), washed twice with 80% ethanol, and eluted in 15 μl nuclease-free water. DNA concentration and fragment-size distribution were assessed using a Qubit dsDNA HS assay (Thermo Fisher Scientific) and an Agilent 4150 TapeStation. The expected fragment-size range was ∼300–800 bp.

#### In vitro transcription and amplified-RNA cleanup

For the BlSq_Anhy and BlSq_H2O2 series, ∼200 ng sBLISS template was used for in vitro transcription (IVT). For the BlSq2_PcH2O2_cT00 and BlSq2_PcH2O2_hT00 series, 100 ng was used when available; 17.6 ng was used for BlSq2_PcH2O2_hT00-3 because of limited template yield. Template was brought to 7.5 μl with nuclease-free water and combined with 12.5 μl IVT master mix containing 8 μl NTP mix, 2 μl 10× reaction buffer, 2 μl T7 enzyme mix, and 0.5 μl RiboSafe RNase inhibitor (in MEGAscript T7 Transcription Kit; Thermo Fisher Scientific). When more than 7.5 μl template was required, the reaction volume was doubled. IVT was performed for 14 h at 37°C with the heated lid set to 70°C.

After IVT, 1 μl DNase I (2 U/μl; Thermo Fisher Scientific) was added and samples were incubated for 15 min at 37°C. Reactions were brought to 30 μl with 9 μl nuclease-free water, mixed with 30 μl RNAClean XP beads (Beckman Coulter), incubated for 10 min at room temperature, and placed on a magnetic stand. Beads were washed twice with 300 μl freshly prepared 80% ethanol and eluted in 7 μl nuclease-free water. Six microliters of eluate was transferred to a new tube for RA3 adaptor ligation, and the remaining 1 μl was used for RNA quantification.

#### RA3 adaptor ligation and reverse transcription

One microliter of 10 μM RA3 adaptor was added to 6 μl purified amplified RNA. Samples were heated at 70°C for 2 min, briefly centrifuged, and placed on ice for at least 1 min. Three microliters of RNA ligation mixture containing 1 μl 10× T4 RNA ligase buffer, 1 μl RNaseOUT recombinant ribonuclease inhibitor (Thermo Fisher Scientific), and 1 μl T4 RNA ligase 2, truncated (200 U/μl; NEB), was added. Ligation was performed at 25°C for 2 h without a heated lid, after which samples were placed on ice.

Reverse transcription was initiated by adding 2 μl of 10 μM reverse transcription primer on ice, followed immediately by 13 μl RT master mix containing 1 μl nuclease-free water, 5 μl 5× RT buffer, 1 μl 25 mM dNTPs, 2 μl 100 mM DTT, 2 μl RNaseOUT recombinant ribonuclease inhibitor (Thermo Fisher Scientific), and 2 μl SuperScript IV reverse transcriptase (Thermo Fisher Scientific). No separate heat-annealing step was performed after the addition of the reverse transcription primer. The 25-μl reaction was incubated at 50°C for 50 min and 80°C for 10 min and then held at 4°C.

#### Library indexing, PCR amplification, cleanup, and sequencing

For library indexing, 20 μl of the selected 10 μM RPIX primer, 135 μl nuclease-free water, 20 μl 10 μM RP1 common primer, and 200 μl NEBNext Ultra II Q5 Master Mix (NEB) were added to the 25-μl cDNA reaction, giving a final volume of 400 μl. Each reaction was divided into eight 50-μl aliquots. Libraries from the BlSq_Anhy and BlSq_H_2_O_2_ series were amplified using the following program: 98°C for 30 s; 8 cycles of 98°C for 10 s, 60°C for 30 s, and 65°C for 45 s; 65°C for 10 min; hold at 12°C. Libraries from the BlSq2_PcH2O2_cT00 and BlSq2_PcH2O2_hT00 series were amplified for 12 cycles using the same cycling conditions. The eight aliquots belonging to each library were pooled after amplification.

The pooled 400-μl PCR product was subjected to double-sided size selection. First, 180 μl AMPure XP beads (0.45×) were added to bind fragments larger than ∼600–700 bp. After magnetic separation, the ∼580-μl supernatant was transferred to a new tube, and 120 μl additional beads (0.30× relative to the original PCR volume) were added. After incubation and magnetic separation, the supernatant was discarded and the beads were washed twice with 700 μl of 80% ethanol. DNA was eluted in 51 μl nuclease-free water, and 50 μl was recovered.

For the second cleanup, 40 μl AMPure XP beads (0.8x) were added to the 50-μl eluate. Beads were washed twice with 150 μl 80% ethanol, and DNA was eluted in 20 μl nuclease-free water. Eighteen microliters of the final library was recovered. Library concentration and size distribution were assessed using a Qubit Fluorometer (Thermo Fisher Scientific) and an Agilent 4150 TapeStation. Final libraries were adjusted to 5 ng/μl, and 5 μl of each library was pooled. Sequencing was performed by BGI on a DNBSEQ-G400 platform using 100-bp single-end reads.

#### Oligonucleotides and adaptor sequences

The oligonucleotides used for sBLISS library preparation were based on the sBLISS protocol of Bouwman et al (2020). All sequences are shown in the 5′ to 3′ direction. N denotes a random nucleotide, /5Phos/ denotes 5′ phosphorylation, /5rApp/ denotes 5′ adenylation, and /3SpC3/ denotes a 3′ C3 spacer.

##### Common oligonucleotides

The RA3 adaptor (Table 1) was ordered at 100-nmol scale with 5′ adenylation and a 3′ C3 spacer. The RT and RP1 primers were ordered at 50-nmol scale. These oligonucleotides were resuspended in TE buffer to 100 μM. The RPIX primers were OPC-purified, ordered at 50-nmol scale, and resuspended in TE buffer to 100 μM.

**Table 1.** Common oligonucleotides for sBLISS library preparation.

Oligonucleotide	Sequence
RA3 adaptor	/5rApp/ TGG​AAT​TCT​CGG​GTG​CCA​AGG /3SpC3/
RT primer	GCC​TTG​GCA​CCC​GAG​AAT​TCC​A
RP1 common primer	AAT​GAT​ACG​GCG​ACC​ACC​GAG​ATC​TAC​ACG​TTC​AGA​GTT​CTA​CAG​TCC​GA
RPIX indexed primer	CAA​GCA​GAA​GAC​GGC​ATA​CGA​GAT [6-nt index] GTG​ACT​GGA​GTT​CCT​TGG​CAC​CCG​AGA​ATT​CCA

##### sBLISS SB adaptors

Thirty-six pairs of HPLC-purified adaptor oligonucleotides (Table 2) were ordered from Eurofins at 50-nmol scale and resuspended in TE buffer to 100 μM. Each adaptor contained an 8-nt UMI and an 8-nt sample barcode (SB).

**Table 2.** sBLISS SB adaptors for sBLISS library preparation.

SB ID	Upper oligo	Upper SB	Lower oligo	Lower SB (rev. comp.)
SB01	sB01_upper	CGTGTGAG	sB01_lower	CTCACACG
SB02	sB02_upper	GACACGAG	sB02_lower	CTCGTGTC
SB03	sB03_upper	ATGTGGAG	sB03_lower	CTCCACAT
SB04	sB04_upper	CTAACTCA	sB04_lower	TGAGTTAG
SB05	sB05_upper	GCTTGTCA	sB05_lower	TGACAAGC
SB06	sB06_upper	AGCCATCA	sB06_lower	TGATGGCT
SB07	sB07_upper	CTAACGAG	sB07_lower	CTCGTTAG
SB08	sB08_upper	GCTTGGAG	sB08_lower	CTCCAAGC
SB09	sB09_upper	TAGGTGAG	sB09_lower	CTCACCTA
SB10	sB10_upper	GAATCCGA	sB10_lower	TCGGATTC
SB11	sB11_upper	ATTCGCGA	sB11_lower	TCGCGAAT
SB12	sB12_upper	TCCGACGA	sB12_lower	TCGTCGGA
SB13	sB13_upper	GAATCTCG	sB13_lower	CGAGATTC
SB14	sB14_upper	ATTCGTCG	sB14_lower	CGACGAAT
SB15	sB15_upper	GAATCATT	sB15_lower	AATGATTC
SB16	sB16_upper	ATTCGATT	sB16_lower	AATCGAAT
SB17	sB17_upper	CGGATACT	sB17_lower	AGTATCCG
SB18	sB18_upper	GACTTCGT	sB18_lower	ACGAAGTC
SB19	sB19_upper	GTCACCGT	sB19_lower	ACGGTGAC
SB20	sB20_upper	TCACATCT	sB20_lower	AGATGTGA
SB21	sB21_upper	ACAGACGC	sB21_lower	GCGTCTGT
SB22	sB22_upper	ACGCTCTA	sB22_lower	TAGAGCGT
SB23	sB23_upper	ACTCATAC	sB23_lower	GTATGAGT
SB24	sB24_upper	AGACAGAC	sB24_lower	GTCTGTCT
SB25	sB25_upper	CACAGCTG	sB25_lower	CAGCTGTG
SB26	sB26_upper	CAGAGTAC	sB26_lower	GTACTCTG
SB27	sB27_upper	CGAGTCTC	sB27_lower	GAGACTCG
SB28	sB28_upper	CGCGAGTA	sB28_lower	TACTCGCG
SB29	sB29_upper	CTGTAGCT	sB29_lower	AGCTACAG
SB30	sB30_upper	GTCGTATA	sB30_lower	TATACGAC
SB31	sB31_upper	TAGTCATC	sB31_lower	GATGACTA
SB32	sB32_upper	TCGATATC	sB32_lower	GATATCGA
SB33	sB33_upper	TCTCGAGC	sB33_lower	GCTCGAGA
SB34	sB34_upper	TGCGATCG	sB34_lower	CGATCGCA
SB35	sB35_upper	TGTACAGT	sB35_lower	ACTGTACA
SB36	sB36_upper	TGTGTAGC	sB36_lower	GCTACACA

Upper oligonucleotide (75 nt): CGATTGAGGCCGGTAATACGACTCACTATAGGGGTTCAGAGTTCTACAGTCCGACGATCNNNNNNNN [SB].

Lower oligonucleotide (77 nt): /5Phos/ [reverse complement of SB] NNNNNNNNGATCGTCGGACTGTAGAACTCTGAACCCCTATAGTGAGTCGTATTACCGGCCTCAATCGAA.

The lower strand oligonucleotides were phosphorylated at their 5′ ends before annealing. The SB sequences used in the upper and lower strands are listed in Table 2.

##### RPIX indexed primers

The RPIX primers (Table 3) contained the common sequence shown in Table 1 and one of the 6-nt index sequences listed in Table 3. Each complete RPIX primer was 63 nt long.

**Table 3.** RPIX indexed primers for sBLISS library preparation.

RPIX primer	6-nt index	RPIX primer	6-nt index	RPIX primer	6-nt index
RPI01	ATCACG	RPI13	AGTCAA	RPI25	ATCAGT
RPI02	CGATGT	RPI14	AGTTCC	RPI26	GCTCAT
RPI03	TTAGGC	RPI15	ATGTCA	RPI27	AGGAAT
RPI04	TGACCA	RPI16	CCGTCC	RPI28	CTTTTG
RPI05	ACAGTG	RPI17	GTAGAG	RPI29	TAGTTG
RPI06	GCCAAT	RPI18	GTCCGC	RPI30	CCGGTG
RPI07	CAGATC	RPI19	GTGAAA	RPI31	ATCGTG
RPI08	ACTTGA	RPI20	GTGGCC	RPI32	TGAGTG
RPI09	GATCAG	RPI21	GTTTCG	RPI33	CGCCTG
RPI10	TAGCTT	RPI22	CGTACG	RPI34	GCCATG
RPI11	GGCTAC	RPI23	GAGTGG	RPI35	AAAATG
RPI12	CTTGTA	RPI24	GGTAGC	RPI36	TGTTGG

##### sBLISS SB adaptors and RPIX indexed primers

sBLISS SB adaptors and RPIX indexed primers used for library preparation are shown in Table 4. Sample names are identical to those used in Tables S9 and S10. Each SB adaptor contains an 8-nt UMI and an 8-nt SB, whereas RPIX denotes the 6-nt index primer added during PCR amplification for sequencing-run demultiplexing.

**Table 4.** sBLISS SB adaptors and RPIX indexed primers for sBLISS library preparation.

Sample	SB adaptor	RPIX primer
BlSq_Anhy_T00-1	SB01	RPI01
BlSq_Anhy_T00-2	SB03	RPI02
BlSq_Anhy_T00-3	SB04	RPI03
BlSq_Anhy_T48-1	SB09	RPI04
BlSq_Anhy_T48-2	SB10	RPI05
BlSq_Anhy_T48-3	SB14	RPI06
BlSq_Anhy_R03-1	SB17	RPI07
BlSq_Anhy_R03-2	SB19	RPI08
BlSq_Anhy_R03-3	SB20	RPI09
BlSq_Anhy_R24-1	SB21	RPI10
BlSq_Anhy_R24-2	SB23	RPI11
BlSq_Anhy_R24-3	SB28	RPI12
BlSq_Anhy_R72-1	SB30	RPI13
BlSq_Anhy_R72-2	SB31	RPI16
BlSq_Anhy_R72-3	SB33	RPI23
BlSq_H2O2_T48-1	SB18	RPI15
BlSq_H2O2_R24-1	SB35	RPI17
BlSq_H2O2_R03-1	SB24	RPI18
BlSq_H2O2_R72-1	SB25	RPI22
BlSq2_PcH2O2_cT00-1	SB02	RPI14
BlSq2_PcH2O2_cT00-2	SB11	RPI20
BlSq2_PcH2O2_cT00-3	SB13	RPI25
BlSq2_PcH2O2_hT00-1	SB27	RPI26
BlSq2_PcH2O2_hT00-2	SB29	RPI34
BlSq2_PcH2O2_hT00-3	SB32	RPI35

### sBLISS-seq data processing and analysis

Demultiplexed sBLISS-seq reads were processed based on the blissNP pipeline with several modifications for increased throughput (https://github.com/BiCroLab/blissNP, commit d39eea92c6c5910850c291f9d0453cae39c8d0e6). Reads were filtered to retain those containing sample-specific barcodes and UMIs, allowing a single mismatch in the UMI sequence. Adapter sequences and UMIs were trimmed, and remaining reads were aligned to the haplotype 1 genome using BWA-MEM v0.7.18-r1243-dirty. The haploid reference was used to reduce ambiguous mappings from regions identical between haplotypes that would otherwise be removed by mapping-quality filters. Reads with MAPQ <60 were discarded with SAMtools. Sequence and file manipulation was performed with SeqKit v2.8.2, BEDTools v2.31.1.

UMIs mapping to the same genomic coordinate, or to proximal positions with up to two mismatches, were collapsed to reduce PCR amplification bias, and unique DSB counts were computed per nucleotide position. DSB signal distributions were analyzed relative to gene bodies and TSSs using deepTools, and enrichment was evaluated using HOMER. BED files produced by blissNP were converted to BAM using BEDTools and normalized using bamCoverage (--binSize 10 --effectiveGenomeSize 120403693 --normalizeUsing RPGC). Normalized matrices were generated with computeMatrix (--missingDataAsZero), and profiles and heatmaps were produced using plotProfile and plotHeatmap. Gene annotations, predicted TSSs, and ATAC-seq peaks were integrated into computeMatrix analyses to quantify DSB enrichment across scaled gene bodies and relative to reference points.

### ATAC-seq analysis

ATAC-seq data from a previous study (NCBI GEO accession ID: GSE190481; GSM5724406; GSM5724411) (Miyata et al, 2022) were used to identify open chromatin regions. Adapter sequences were removed using Cutadapt v4.9 (Martin, 2011), and trimmed reads were mapped to the diploid *Po. vanderplanki* genome using Bowtie2 v2.5.4. PCR duplicates were removed using Picard MarkDuplicates v3.1.1, and mitochondrial reads were excluded using BEDTools v2.31.1.

Library complexity was assessed by calculating PCR bottlenecking coefficient metrics using BEDTools and a custom script following ENCODE ATAC-seq guidelines (https://www.encodeproject.org/atac-seq/). Insert-size distribution was calculated and visualized with Picard CollectInsertSizeMetrics. Peaks were called using Genrich v0.6.2 (https://github.com/jsh58/Genrich), and read counts over peaks were obtained using featureCounts. Differential accessibility was analyzed using DESeq2 v1.46.0 (Love et al, 2014). For visualization, BAM files were normalized with bamCoverage (--binSize 20 --effectiveGenomeSize 240403693 --normalizeUsing RPGC--smoothLength 60). Signal matrices were generated using computeMatrix (--missingDataAsZero) and visualized using plotProfile and plotHeatmap.

### Software availability

The software packages used in this study are described in the cited publications. Custom scripts are available from a dedicated GitHub repository at https://github.com/Kikawada-Lab-UT-NARO/Pv11_Diploid_Genome_Paper.

## Supplementary Material

Reviewer comments

## Data Availability

PacBio HiFi sequencing data have been deposited in the DNA Data Bank of Japan under accession DRR537985. DNBSEQ and sBLISS sequencing data have been deposited in the NCBI under BioProject accession PRJNA1377239. The genome assembly and gene predictions are available from Figshare under https://doi.org/10.6084/m9.figshare.30930980 (Yoshida & Kikawada, 2026a). The genomic sBLISS UMI-count data are available from Figshare under https://doi.org/10.6084/m9.figshare.31290736 (Yoshida & Kikawada, 2026b).
