# Supplementary material for: Haplotype-resolved genome of a dry-preservable cell line reveals chromosome evolution and DNA damage
Source: Life Sci Alliance. 2026 Oct 6;9(12):e202603719. doi: 10.26508/lsa.202603719 (PMC13642679; doi:10.26508/lsa.202603719)
Supplement: Supplementary file 1 [file LSA-2026-03719_review_history.pdf]

Haplotype-resolved genome of a dry-preservable cell line reveals chromosome evolution and DNA damage

Yuki Yoshida and Takahiro Kikawada
DOI: https://doi.org/10.26508/lsa.2026003719

Corresponding author(s): Prof. Takahiro Kikawada (National Agriculture and Food Research Organization; The University of Tokyo)

Review Timeline:

Submission Date:	2026-03-28
Editorial Decision:	2026-06-02
Revision Received:	2026-08-10
Editorial Decision:	2026-09-08
Revision Received:	2026-09-14
Accepted:	2026-09-14

Scientific Editor: Tim Fessenden

Transaction Report:

(Note: With the exception of the correction of typographical or spelling errors that could be a source of ambiguity, letters and reports are not edited. The original formatting of letters and referee reports may not be reflected in this compilation.)

June 2, 2026

Re: Life Science Alliance manuscript #LSA-2026-03719-T

Takahiro Kikawada
National Agriculture and Food Research Organization (NARO)

Dear Dr. Kikawada,

Thank you for submitting your manuscript entitled "Haplotype-resolved diploid genome assembly of an anhydrobiotic cell line reveals chromosome evolution and desiccation-associated DNA damage" to Life Science Alliance. We appreciate your patience during the unusually long review period. The manuscript was assessed by expert reviewers, whose comments are appended to this letter.

As you will see, all reviewers appreciated this high resolution genome assembly with experimental data on desiccation. Reviewers 2 and 3 made suggestions to adjust the text to soften certain claims and ensure clarity and readability. Reviewer 1 requested you expand the species comparison to better support the intriguing claim on extreme genome shuffling, currently based on a comparison to Chironomus riparius. We also concur that, if possible, this study would be improved by the addition of ultra-long ONT sequencing as mentioned by this reviewer. We leave this to your discretion. We invite you to submit a revised manuscript that addresses all reviewer comments.

I would be happy to discuss the revision in more detail via email or phone/videoconferencing. Please let me know which option you prefer, if any.

To upload the revised version of your manuscript, please log in to your account: https://lsa.msubmit.net/cgi-bin/main.plex

You will be guided to complete the submission of your revised manuscript and to fill in all necessary information. Please get in touch in case you do not know or remember your login name.

The typical timeframe for revisions is three months. Please note that papers are generally considered through only one revision cycle, so strong support from the referees on the revised version is needed for acceptance.

While you are revising your manuscript, please also attend to the below editorial points to help expedite the publication of your manuscript. Please direct any editorial questions to the journal office. When submitting the revision, please include a letter addressing the reviewers' comments point by point.

Thank you for this interesting contribution to Life Science Alliance. We hope that the comments below will prove constructive as your work progresses, and we are looking forward to receiving your revised manuscript.

Sincerely,

Tim Fessenden
Scientific Editor
Life Science Alliance

A. THESE ITEMS ARE REQUIRED FOR REVISIONS

-- A letter addressing the reviewers' comments point by point.

-- An editable version of the final text (.DOC or .DOCX) is needed for copyediting (no PDFs).

-- High-resolution figure, supplementary figure and video files uploaded as individual files: See our detailed guidelines for preparing your production-ready images, https://www.life-science-alliance.org/authors

-- Summary blurb (enter in submission system): A short text summarizing in a single sentence the study (max. 200 characters including spaces). This text is used in conjunction with the titles of papers, hence should be informative and complementary to the title and running title. It should describe the context and significance of the findings for a general readership; it should be written in the present tense and refer to the work in the third person. Author names should not be mentioned.

-- By submitting a revision, you attest that you are aware of our payment policies found here: https://www.life-science-alliance.org/copyright-license-fee

B. MANUSCRIPT ORGANIZATION AND FORMATTING:

Full guidelines are available on our Instructions for Authors page, https://www.life-science-alliance.org/authors

We encourage our authors to provide original source data, particularly uncropped/-processed electrophoretic blots and spreadsheets for the main figures of the manuscript. If you would like to add source data, we would welcome one PDF/Excel-file per figure for this information. These files will be linked as supplementary "Source Data" files.

IMPORTANT: It is Life Science Alliance policy that if requested, original data images must be made available. Failure to provide original images upon request will result in unavoidable delays in publication. Please ensure that you have access to all original microscopy and blot data images before submitting your revision.

Reviewer #1 (Comments to the Authors (Required)):

1. The manuscript entitled 'Haplotype-resolved diploid genome assembly of an anhydrobiotic cell line reveals chromosome evolution and desiccation-associated DNA damage' is providing a novel, high quality genome assembly of Polypedilum vanderplanki cell line using high quality long reads and short reads technologies to correct and scaffold the assembly. Importantly authors provide extra analyses investigating the interesting biology, both in silico (variant calling, levels of SNV along the chromosomes) and experimentally such as the investigation of location of double strand breaks of the DNA during the trehalose treatment with extra sequencing data. The prepared manuscript offers significant advances as the haplotype-resolved assembly was lacking for this important model for anhydrobiosis resistance.

2.1 The haplotype resolved genome assembly is of high quality thanks to the use of various complementing datasets: PacBio HiFi reads, Illumina short reads and ultralong ONT reads. The data is strongly supportive over here. However, I have a few minor comments on the assembly and annotation and further technical parts:
Page 9, lines 190-191: Why do you mention it over here? There is no previous information on functional protein-coding genes located on Chr 4. This paragraph mostly concern evolution on nucleotide level
Page 11, lines 229-231: This interaction would be visible in the HiC contact map. Neither in the main text, nor in the supplementary data can I find the full HiC map visualising the chromosomes. Please consider adding it.
Page 17, lines 369-372: For researchers not accustomed to this model it may be confusing and problematic to keep track of the strains and datasets. I would suggest adding a (supplementary?) table that summarises the cell lines and what type of data was generated. Possibly alongside the version of assemblies, to easily keep track of what dataset were used and when they were generated.
Page 17, lines 373-374: Fully agree with that and I understand that the main reason this was not achieved for this study were time/funding limitations? The extraction of High Molecular Weight DNA from a cell line should be relatively easy given plenty of material (lab cell line) and lack of exoskeleton to digest in the first please. Can you address this?
Page 17, lines 375-376: What does the coverage of the putative merged region looks like? Do the reads map well to this part of the assembly?
Page 17, line 380: add reference to Figure 2 here.
Page 18, lines 400-401: I may be missing a point here but why this value is unexpected? this suggests an even distribution of haplotype specific genes across the chromosomes. 20-25% in one of 4 chromosomes indicate 75-80% is present on remaining three chromosomes, which gives 20-25% per chromosome (?).
Page 22, lines 500-503: How much did it improve the long read assembly?
Page 24, line 564: what was the fraction of ONT reads removed? Have you checked if retaining shorter reads help with the coverage and reconstructing the more complex parts of the genome assembly? Would be especially interesting to see if there are reads supporting the suggested fusion of chromosomes.

2.2 The Extreme genome shuffling within Chironomini - this part is an interesting comparative genomics analysis and is supported by the data presented. However, this interesting theme of your paper is done on too limited scale. There are several chironomid chromosome level genomes publicly available. It would be very interesting to see if the conservation of chromosome content is present on a broader phylogenetic scale and if the within-chromosome reshuffling of genes is an oddity or a rule in this family. At the moment one may be tempted to draw a conclusion that maybe P. vanderplanki is a special case due to anhydrobiotic abilities. The broader compassion would serve as an intimidate answer.
Another question is why C. riparius was chosen for the comparison, not any other species? Obviously other Polypedilum would be better but these are not available.
Page 15, lines 229-330 (ii): with two genomes compared this is overly confident statement. Please consider my previous comment on that.
Page 16, line 336 (on the phrase higher eukaryotes): Please do not use such wording. Diptera are very complex, evolutionary

advanced and successful group. There are single cell eukaryotes with large genome sizes, plentiful of repeats an/or complex genome architecture. Surely you wouldn't call them "higher" species in comparison, although their genomes are extremely difficult to assemble (some undoable with current technology). You can refer here to larger genome sizes or more generally to larger/more complex and therefore difficult to assemble genomes.

3. Minor issues:
Page 3, line 64 (on Yoshida et al. 2022b): This is the first citation of Yoshida et al. 2022 so it should be changed to a, preferably.
Page 4, lines 75 and 83: The Latin names e.g. Chironomus riparius and P. vanderplanki should be in italics. Please check across the manuscript. Also page 11, line 222; page 16, line 356.
Page 6, lines 114-115: how about the grey dots? No telomeric repeats detected?
Page 7, lines 137-138: could you add the values of BUSCO completness here for each haplotype?
Page 14, lines 304-305: (consistent with patterns reported in mammalian cell lines.) could you add citation over here?
Page 16, line 357: (Repetitive sequences are the hallmark of genomic instability) could you add citation over here?
Page 19, line 430: I would suggest citing here this paper as one of the most comprehensive analyses of post-stress response: DOI: 10.1126/science.adl0799
Page 21, line 479: please consider adding also these citations: https://pubmed.ncbi.nlm.nih.gov/31340759/ ; DOI: 10.1126/science.adl0799
Page 22, line 520: reverse the order of citations (neither alphabetical nor chronological now).

Reviewer #2 (Comments to the Authors (Required)):

The manuscript presents a haplotype-resolved diploid genome assembly for Pv11, an anhydrobiosis-capable cell line derived from the chironomid midge Polypedilum vanderplanki. The authors argue that previous genomic resources were limited by haplotype switching, consensus errors, local misassemblies, and the use of mixed insect and cell-line DNA. To address this, they generated PacBio HiFi data from a monoclonal Pv11 isolate and combined these reads with Hi-C and ONT data. The final assembly contains eight chromosome-level scaffolds. Using this assembly, the authors revisit chromosome 4, previously proposed as unusually divergent. They confirm elevated Chr4 heterozygosity with both read-based and assembly-alignment-based SNV calls, then link this divergence to gene-proximal variation, missense variants, haplotype-specific genes, and higher Ka and Ks values. Population-level analyses of independently maintained Pv11 cultures do not support ongoing Chr4-specific drift in culture. The paper also identifies two candidate telomeric repeat units, leaves centromere structure unresolved, and suggests a possible Chr2 and Chr3 fusion. Comparative synteny with Chironomus riparius indicates extensive within-chromosome gene-order reshuffling while preserving broad chromosomal identity. Finally, the authors use sBLISS to map DNA double-strand breaks during the anhydrobiosis cycle. Rehydration samples are treated cautiously because of high cell death. The main DNA damage claim is therefore based on trehalose pretreatment, where DSBs concentrate near CAGE-defined transcription start sites, accessible chromatin, and highly expressed genes.

General assessment

This manuscript provides a useful genomic resource for Pv11 cells and uses it to revisit haplotype structure, chromosome evolution, and DNA double-strand break localization during trehalose pretreatment. The strongest parts are the assembly work and the descriptive analysis of chromosome 4 heterozygosity. The sBLISS result showing promoter-proximal DSB enrichment is also convincing, especially because it is integrated with CAGE-seq and ATAC-seq. The main weakness is that several broader biological interpretations go beyond what the current data directly test. These include the proposed causes of Chr4 divergence, the possible Chr2 and Chr3 fusion, telomere and centromere evolution, and the model that trehalose pretreatment both generates DSBs and activates protective or repair pathways. I would recommend revision before publication. The manuscript does not need to be reframed entirely, but it should distinguish more clearly between well-supported results, plausible interpretations, and hypotheses for future work.

Specific comments

The claim that the authors have produced an improved haplotype-resolved Pv11 assembly is well supported. The authors could make the assembly evaluation more transparent in the main text. For example, a table comparing v5.2, v6.0, v6.1, v6.2, and v7.0 would help readers judge the improvement. This table should include contig number, scaffold number, total assembly size, N50 values, number of gaps, BUSCO or compleasm scores, Merqury QV, and whether each assembly is haplotype-resolved. I would suggest that the authors should avoid language that could imply a complete or near-complete assembly. The main text states that six gaps remain in chromosome-scale scaffolds and that complex terminal and centromeric regions remain unresolved. That is certainly acceptable, but the framing should consistently describe v7.0 as a high-quality draft diploid assembly rather than a complete reference.

The manuscript convincingly shows elevated heterozygosity on Chr4 using two independent approaches, DeepVariant on haplotype-separated HiFi reads and Syri haplotype-to-haplotype alignment. Both approaches show increased SNV density on Chr4. This makes the descriptive claim strong. The downstream claim that Chr4 divergence affects gene regions and coding

sequences is moderately well supported. Chr4 shows enrichment of upstream and downstream variants, more missense variants, lower amino acid identity, more haplotype-specific genes, and elevated Ka and Ks. The expression-stratified Ka result is also consistent with stronger constraint on highly expressed genes. The interpretation that Chr4 experiences elevated mutation input or distinct selective constraints is plausible, but the current data do not separate these mechanisms. The manuscript's "and/or" phrasing is appropriate and should be retained. The authors should explicitly separate three claims: Chr4 is highly heterozygous, Chr4 variation affects gene-proximal and coding regions, and the mechanism may involve mutation rate, selection, or historical demography. The first claim is strong. The second is moderate to strong. The third remains unresolved. I would suggest that the discussion avoids implying that relaxed selection is the primary explanation unless additional tests are added. A sentence such as "These data cannot distinguish increased mutation input from altered selective constraint" would improve balance.

The comparison with Chironomus riparius supports extensive intrachromosomal reshuffling while preserving broad chromosome identity. This claim is supported by MCScan synteny blocks and bidirectional best-hit ortholog assignments. The analysis is useful, but a two-species comparison cannot fully establish the history, rate, or direction of rearrangements. The title "Extreme genome shuffling within Chironomini" may overstate the inference. Consider changing the section title to "Extensive intrachromosomal rearrangement within Chironomini." The authors should also clarify whether reduced macrosynteny reflects true rearrangement, divergence affecting ortholog detection, assembly differences, or a combination. Adding another high-quality chironomid genome, if available, would help polarize rearrangements. If no additional genome is suitable, the authors could add statistical support for synteny block depletion or rearrangement enrichment by chromosome.

Minor textual suggestions

Use "extensive" rather than "extreme" for genome shuffling.

Use "candidate telomeric repeats" throughout unless FISH or another localization assay is added.

In the abstract, reduce forward-looking claims about engineering stress-resilient cells unless that is tied more directly to demonstrated results.

Where the manuscript presents models, label them as models. This is especially important for the proposed link among trehalose, hyperosmotic stress, transcriptional activation, DNA repair, and promoter-proximal DSB accumulation.

Reviewer #3 (Comments to the Authors (Required)):

This paper describes the sequencing, assembly and analysis of a haplotype-resolved genome of the cell line derived from the desiccation-tolerant chironomid Polypedilum vanderplanki. The authors also analysed haplotype diversity, heterozygosity patterns and haplotype-specific gene content, and suggest that specific regions are hotspots for DNA DSBs during desiccation-inducing experiments.
While I am not an expert in all the assembly technical details, I think this is a well-written and original paper, which adds significant data to the general knowledge of desiccation, with conclusions well supported by the data.

I attach some minor comments which I suggest the Authors to address, to make the text clearer and easier to read:

Throughout the text
Some minor formatting issues, e.g. species names not in italics (e.g. lines 75, 83, but others in the text), missing spaces between two words (e.g. lines 560, 690), close parenthesis missing at line 768.
The Supplementary material was difficult to link to the text as the files names were not clear, making it difficult to find the correct file, This might be due to the submission format, but please make sure that in the final version each file is names accordingly, e.g. name "Suppl. Table 1" as such and not "4588_0_unknown_upload_747404_tcm409".
The consistent colour-coding across the figures and the very clear formatting of the Suppl. Tables make the text clear and easy to read, thank you.
I would suggest writing Latin text in italics, e.g. Latin words like in situ, versus, locus/loci, etc.

Abstract
Lines 21-22: Anhydrobiosis has developed in non-metazoans as well, while here the authors only mention aquatic invertebrates. While apparently a minor point, I suggest mentioning it, otherwise it looks too unique (e.g. say "in many organisms, and in some lines of aquatic invertebrates" or similar).

Keywords
The Authors mention sBLISS but this should be defined here, so the Keywords could be "Chironomid, Pv11 cells, anhydrobiosis, haplotype-resolved diploid genome, DNA double-strand breaks (DNA DSBs), in-suspension breaks labeling in situ and sequencing (sBLISS)".

Introduction
Line 58, "A distinctive advantage of P. vanderplanki is the availability of an anhydrobiosis-capable cultured cell line, Pv11 (Nakahara et al. 2010)": I suggest including a few words about where the cell line was isolated from, just before the reference (e.g. was the cell line isolated form a single animal? Single tissue? Just to put this in context.
Lines 120-125: if possible, I would suggest adding a figure in the text to visualise this part (the gap in Chr 4), or add this to Fig. 1A.
The Introduction is currently mainly focused on the genome assembly, but I would add a short paragraph on the desiccation aspects (background knowledge, the fact that desiccation can be induced by trehalose) so that the results in the section "Double-strand breaks are enriched around transcription start sites" become clearer.

Results
At the beginning of the results, some basic summary statistics for the genome assemblies might be useful in the text (e.g. chromosome length, etc.).

Discussion
The Authors used trehalose preconditioning to trigger DNA DSBs, but this only becomes clear in the discussion I would suggest toad a short paragraph in the Introduction to make a clearer link in the Discussion.
It would be interesting to discuss a little bit more about horizontally transferred genes, as currently they are only mentioned in the text.

Supplementary text
I would suggest integrating the Supplementary text into the main text of the paper, as some of these analyses are as interesting as the rest of the paper (and leave the Suppl Figures, Tables and their legends as supplementary material).

Figures
All Figures: Remove the titles e.g. "Figure 1" from each figure (as it is already in the figure legend).
All Figures: I realise there is lots of data in the figures, but consider making the writing larger wherever possible, to make the figures easier to read. E.g. the y axes labels of the Coverage graphs in Figure 1B could be slightly larger. Same for e.g. Figure 2, etc.
Figure 1A: the symbols for the "Unplaced contigs" is not very clear in the figure, consider making it darker/more visible.

Reply to Reviewers' comments

We thank the editor and reviewers for their constructive comments. In the revised manuscript, we have strengthened the evaluation and framing of the haplotype-resolved Pv11 genome assembly, expanded the comparative synteny analysis by including *Propsilocerus akamusi* as a phylogenetically more distant chironomid comparator, and clarified the interpretation of Chr4 divergence. We have also added a genome-wide Hi-C contact map, revised the discussion of candidate telomeric repeats and the possible Chr2–Chr3 fusion, and distinguished direct observations from working models in the analysis of trehalose-associated DNA double-strand breaks. In addition, the Supplementary Methods now provide experimentally detailed sBLISS procedures and sample-specific adaptor and index information, and we have corrected formatting, terminology, and figure-labeling issues throughout the manuscript. All changes are described point by point below.
Because both *Polypedilum* and *Propsilocerus* are discussed in the revised manuscript, we use the abbreviations *Po*. and *Pr*., respectively, after their first full mention.

Reviewer #1 (Comments to the Authors (Required)):

1. The manuscript entitled 'Haplotype-resolved diploid genome assembly of an anhydrobiotic cell line reveals chromosome evolution and desiccation-associated DNA damage' is providing a novel, high quality genome assembly of *Polypedilum vanderplanki* cell line using high quality long reads and short reads technologies to correct and scaffold the assembly. Importantly authors provide extra analyses investigating the interesting biology, both in silico (variant calling, levels of SNV along the chromosomes) and experimentally such as the investigation of location of double strand breaks of the DNA during the trehalose treatment with extra sequencing data. The prepared manuscript offers significant advances as the haplotype-resolved assembly was lacking for this important model for anhydrobiosis resistance.
We thank the reviewer for their thorough evaluation of our manuscript and hope that the responses below address each point satisfactorily.

2.1 The haplotype resolved genome assembly is of high quality thanks to the use of various complementing datasets: PacBio HiFi reads, Illumina short reads and ultralong ONT reads. The data is strongly supportive over here. However, I have a few minor comments on the assembly and annotation and further technical parts:

Page 9, lines 190-191: Why do you mention it over here? There is no previous information on functional protein-coding genes located on Chr 4. This paragraph mostly concern evolution on nucleotide level

The sentence about the two additional LEA genes was not well placed in a paragraph focused on haplotype-level coding divergence. We removed this sentence from the Results.

Page 11, lines 229-231: This interaction would be visible in the HiC contact map. Neither in the main text, nor in the supplementary data can I find the full HiC map visualising the chromosomes. Please consider adding it.

We have generated a genome-wide contact map (Fig. 1C). Although an interaction between Chr2 and Chr3 was not apparent in this contact map, we consider the map itself important for evaluating the genome assembly and have therefore incorporated it into the figure.

Page 17, lines 369-372: For researchers not accustomed to this model it may be confusing and problematic to keep track of the strains and datasets. I would suggest adding a (supplementary?) table that summarises the cell lines and what type of data was generated. Possibly alongside the version of assemblies, to easily keep track of what dataset were used and when they were generated.

We apologize for the confusion. Supplementary Table 1 contains information on sequencing data generated in this study and data obtained from our previous studies. We have added the origin of each dataset to clarify which data were generated in the present study. Similarly, Supplementary Table 2 contains assembly statistics for all genome assemblies generated in this study.

Page 17, lines 373-374: Fully agree with that and I understand that the main reason this was not achieved for this study were time/funding limitations? The extraction of High Molecular Weight DNA from a cell line should be relatively easy given plenty of material (lab cell line) and lack of exoskeleton to digest in the first please. Can you address this?

At the time of genome assembly, we considered the 50-kb-filtered ONT reads sufficient because Hi-C data were also available. Although the resulting assembly contained several regions that would require further refinement, including telomeres, the putative Chr2–Chr3 fusion, and multiple gaps across the genome, resolving these regions to produce a complete genome was beyond the scope of the present study. We plan to address these issues in future work.

Page 17, lines 375-376: What does the coverage of the putative merged region looks like? Do the reads map well to this part of the assembly?

We thank the reviewer for raising this point. To examine read support around the putative Chr2–Chr3 junction, we generated an artificial Chr2_Hap1–Chr3_Hap1 concatenation separated by 50 Ns and mapped the PacBio HiFi and >50-kb ONT reads to this sequence. Reads mapped to the regions flanking both scaffold ends, indicating that these regions were represented in the sequencing data. However, no individual read or consistent set of alignments provided an unambiguous continuous connection across the putative junction. Alignments near the scaffold ends frequently terminated or were soft-clipped, which could reflect unresolved repetitive or structurally complex sequence, incomplete terminal assembly, or a true chromosome junction.

In addition, the genome-wide Hi-C contact map did not show conspicuous contact enrichment between the relevant ends of Chr2 and Chr3. Thus, the current read-mapping and Hi-C data neither confirm nor exclude a Chr2–Chr3 fusion. Because mapping to an artificially concatenated sequence did not resolve a physical junction, we chose not to present this inspection as a separate figure and instead revised the manuscript to state the limitation explicitly. Additional matched ultra-long ONT reads and cytogenetic validation will be required to resolve this question.

The following text was added to the Results:

Chromosomes 2 and 3 were also connected in the assembly graph, raising the possibility that these scaffold ends represent an unresolved Chr2–Chr3 junction. However, the genome-wide Hi-C map did not provide clear independent support for contact between the relevant Chr2 and Chr3 ends, and the available long reads did not resolve an unambiguous junction. Thus, the current data neither confirm nor exclude a Chr2–Chr3 fusion. Resolving this possibility would require additional ultra-long-read sequencing and cytogenetic validation.

The following text was added to the Discussion:

The assembly graph connected Chr2 and Chr3, but neither the genome-wide Hi-C map nor the available long-read alignments resolved an unambiguous junction. Although reads mapped to the regions flanking both scaffold ends, no read provided a definitive bridge between them. The absence of detectable Unit_A or Unit_B sequences at these scaffold ends could also reflect incomplete assembly of terminal repeats. Thus, the current data neither confirm nor exclude a Chr2–Chr3 fusion.

[Figure removed by editorial staff per authors' request].

Page 17, line 380: add reference to Figure 2 here.

Fixed accordingly.

Page 18, lines 400-401: I may be missing a point here but why this value is unexpected? this suggests an even distribution of haplotype specific genes across the chromosomes. 20-25% in one of 4 chromosomes indicate 75-80% is present on remaining three chromosomes, which gives 20-25% per chromosome (?).

Chromosome 4 is approximately 14 Mb in length, corresponding to 11.9% of the genome (14/120 Mb), and harbors 14.3% of the annotated transcripts (2,818/19,747). We have added these values to the Results section, as shown below.

> In addition, 6.6% of Hap1 genes and 2.9% of Hap2 genes were not recovered in the opposite haplotype. Approximately 20–25% of these haplotype-specific genes mapped to Chr4 (25.9% for haplotype 1 and 21.7% for haplotype 2), although Chr4 spans only 11.9% of the genome and harbors 14.3% of the transcripts.

Page 22, lines 500-503: How much did it improve the long read assembly?

The raw NextDenovo assembly had a Compleasm score of C:96.56% [S:95.56%; D:1.00%], F:0.40%, M:3.04%, and a Merqury QV of 33.11. After NextPolish, the assembly had a Compleasm score of C:96.44% [S:95.37%; D:1.07%], F:0.40%, M:3.17%, and a Merqury QV of 39.17. Thus, NextPolish improved the QV score but did not improve the Compleasm score, which decreased slightly by 0.12 percentage points. Neither assembly exceeded the quality of the Pv11 v7.0 diploid assembly. These results have been added to Supplementary Table

S2.

Page 24, line 564: what was the fraction of ONT reads removed?

After filtering for reads longer than 50 kb, 38,612 of 290,835 ONT reads were retained, corresponding to 13.27% of the original read set. To make this clearer for readers, we have added the following text to the Methods section.

> Only ONT reads longer than 50 kb were used (13.27% of the reads were retained).

Have you checked if retaining shorter reads help with the coverage and reconstructing the more complex parts of the genome assembly? Would be especially interesting to see if there are reads supporting the suggested fusion of chromosomes.

We did not rerun the assembly using the excluded ONT reads shorter than 50 kb. Instead, as described in our response above, we evaluated the available PacBio HiFi and >50-kb ONT reads around the putative Chr2–Chr3 junction. Reads mapped to both flanking scaffold ends, but none provided an unambiguous continuous bridge. Because the excluded R9-chemistry reads were shorter and more error-prone, we did not expect them to resolve this structurally complex region reliably. We therefore revised the manuscript to state that the current data neither confirm nor exclude a Chr2–Chr3 fusion and that additional ultra-long-read sequencing and cytogenetic validation will be required.

- 2.2 The Extreme genome shuffling within Chironomini - this part is an interesting comparative genomics analysis and is supported by the data presented. However, this interesting theme of your paper is done on too limited scale. There are several chironomid chromosome level genomes publicly available. It would be very interesting to see if the conservation of chromosome content is present on a broader phylogenetic scale and if the within-chromosome reshuffling of genes is an oddity or a rule in this family. At the moment one may be tempted to draw a conclusion that maybe *P. vanderplanki* is a special case due to anhydrobiotic abilities. The broader compassion would serve as an intimidate answer.
- Another question is why *C. riparius* was chosen for the comparison, not any other species? Obviously other *Polypedilum* would be better but these are not available.
- Page 15, lines 229-330 (ii): with two genomes compared this is overly confident statement. Please consider my previous comment on that.

C. riparius was initially selected because it provides a published chromosome-level genome from Chironominae, allowing comparison with Pv11 within the same subfamily. We recognize that additional chromosome-level assemblies have recently become available for

Chironomus striatipennis and *Chironomus kiiensis*; however, these genomes are not yet associated with published manuscripts, and we therefore chose not to include them in the present analysis. Although a chromosome-level genome assembly of *Chironomus tentans* has been published, including multiple genomes from a single genus could bias the comparative analysis toward Chironomus-specific patterns. We therefore used *C. riparius* for comparison with a close relative.

However, we agree that the original comparison between Pv11 and *C. riparius* alone was insufficient to determine whether the observed intrachromosomal reshuffling was restricted to a pairwise comparison within Chironominae. We therefore extended the analysis to *Propsilocerus akamusi,* a phylogenetically more distant chironomid outside Chironominae. In the revised manuscript, *Pr. akamusi* is treated as a member of Orthocladiinae *sensu lato*, although it has also been classified in Prodiamesinae.

We observed fragmented synteny blocks between Pv11 and *Pr. akamusi*, similar to the pattern observed between Pv11 and *C. riparius* (new Fig. 3A). These results indicate that extensive intrachromosomal rearrangement is not restricted to the comparison between Pv11 and *C. riparius*, both members of Chironominae, but is also evident in comparisons spanning a deeper subfamily-level divergence.

Thus, we have revised the Results, Discussion, Methods, and Figure 3 to emphasize the phylogenetic scope of this comparison. At the same time, we now state explicitly that the present three-species analysis does not establish the ancestral chromosome arrangement, direction, or rate of individual rearrangements, and does not demonstrate that the pattern is universal throughout Chironomidae. Further analyses using additional chromosome-level assemblies, such as *Diamesa*, *Smittia*, and other *Chironomus* species, may provide deeper insight into Chironomidae genome evolution.

The following text was added to the Results:

Cytological analyses using salivary gland polytene chromosomes have suggested extensive intrachromosomal rearrangement, possibly mediated by repetitive elements. Taking advantage of the highly contiguous Pv11 genome assembly, we compared gene-level synteny with *C. riparius* from the same subfamily, Chironominae, and *Propsilocerus akamusi* from a different subfamily, Orthocladiinae *sensu lato*. Small synteny blocks were detected across all four chromosomes between Pv11 and *C. riparius* but were extensively rearranged within chromosomes (Fig. 3A). Similarly fragmented synteny was observed in comparisons involving *Pr. akamusi*, including both Pv11-to-*Pr. akamusi* and *C. riparius*-to-*Pr. akamusi*. Thus, extensive intrachromosomal rearrangement was not

restricted to the comparison between the two Chironominae species but was also evident in comparisons spanning a deeper subfamily-level divergence.

The following text was added to the Discussion:

The diploid reference also strengthens comparative analyses of chromosome evolution in chironomids. Cytogenetic studies of Chironomus have documented extensive inversion polymorphism and structural chromosome variation (Kiknadze, 2008), and some chromosomal breakpoints colocalize with transposable elements or tandemly repetitive DNA (Bovero et al, 2002, Michailova et al, 2009). To examine chromosome evolution across different phylogenetic depths within Chironomidae, we compared gene-level synteny among Pv11 cells, derived from *Po. vanderplanki*, *C. riparius*, and *Pr. akamusi*. *Polypedilum vanderplanki* and *C. riparius* belong to Chironominae, whereas *Pr. akamusi* is treated here as a member of Orthocladiinae sensu lato, although it has also been classified within Prodiamesinae (Semenchenko et al, 2025, Xu et al, 2026).

All three pairwise comparisons showed highly fragmented synteny blocks. In the comparison between Pv11 and *C. riparius*, gene order was extensively reshuffled within chromosomes, while broad chromosome identity was retained. Comparisons involving the more distantly related *Pr. akamusi* showed similarly fragmented synteny, indicating that this pattern is not restricted to a single species pair or to divergence within Chironominae.

Nevertheless, this three-species comparison does not establish that extensive intrachromosomal reorganization is universal across Chironomidae, nor does it resolve the ancestral chromosome arrangement or the direction and rate of individual rearrangements. Differences in sequence divergence, assembly quality, gene annotation, and ortholog detection may also contribute to the apparent fragmentation of synteny blocks. Additional chromosome-level genomes representing other chironomid subfamilies will therefore be required to determine how broadly this pattern occurs across the family.

Page 16, line 336 (on the phrase higher eukaryotes): Please do not use such wording. Diptera are very complex, evolutionary advanced and successful group. There are single cell eukaryotes with large genome sizes, plentiful of repeats an/or complex genome architecture. Surely you wouldn't call them "higher" species in comparison, although their genomes are extremely difficult to assemble (some undoable with current technology). You can refer here

to larger genome sizes or more generally to larger/more complex and therefore difficult to assemble genomes.

We apologize for the confusion. We have removed the sentence referring to higher eukaryotes, as it does not provide direct support for unresolved assembly errors in the Pv11 genome.

3. Minor issues:

Page 3, line 64 (on Yoshida et al. 2022b): This is the first citation of Yoshida et al. 2022 so it should be changed to a, preferably.

We have checked the reference format instructions, which state that references should be ordered alphabetically. The two relevant citations are Yoshida Y, Satoh T, et al. 2022 and Yoshida Y, Shaikhutdinov N, et al. 2022. Therefore, Yoshida Y, Satoh T, et al. 2022 should be cited in the text as Yoshida et al. 2022a, as currently formatted in the manuscript.

Page 4, lines 75 and 83: The Latin names e.g. *Chironomus riparius* and *P. vanderplanki* should be in italics. Please check across the manuscript. Also page 11, line 222; page 16, line 356.

Fixed accordingly.

Page 6, lines 114-115: how about the grey dots? No telomeric repeats detected?

We thank the reviewer for identifying this error. Grey dots indicate chromosome ends at which no telomeric repeats were detected. We have added this explanation to the text:

> Colored dots at chromosome ends indicate telomeric repeat units (Unit_A, red; Unit_B, yellow; neither detected, grey).

Page 7, lines 137-138: could you add the values of BUSCO completness here for each haplotype?

We have added the following text to clarify this point.

> Gene prediction with BRAKER3 identified 30,930 genes (Hap1: 15,790; Hap2: 15,140), with 97.42% compleasm completeness (Hap1: 96.13%; Hap2: 95.74%).

Page 14, lines 304-305: (consistent with patterns reported in mammalian cell lines.) could you add citation over here?

Fixed accordingly (added Bouwman et al.)

Page 16, line 357: (Repetitive sequences are the hallmark of genomic instability) could you

add citation over here?

After further review, we found that the statement “Repetitive sequences are the hallmark of genomic instability” did not fit the intended context. We have therefore removed this sentence.

Page 19, line 430: I would suggest citing here this paper as one of the most comprehensive analyses of post-stress response: DOI: 10.1126/science.adl0799

We have added Anoud et al. (eLife, 2024) and Li et al. (Science, 2024) as examples of studies focusing on DNA repair responses to genotoxic stress in tardigrades.

Page 21, line 479: please consider adding also these citations: https://pubmed.ncbi.nlm.nih.gov/31340759/ ; DOI: 10.1126/science.adl0799

Respectfully, Kamilari et al. did not analyze the cross-talk between radiation responses and anhydrobiosis, but instead focused on anhydrobiosis in *Echiniscoides* cf. *sigismundi* and *Richtersius* cf. *coronifer*. We therefore considered that this reference was not well suited to the context of this sentence. We have instead added Li et al. (Science, 2024) to this section.

Page 22, line 520: reverse the order of citations (neither alphabetical nor chronological now).

Fixed accordingly.

Reviewer #2 (Comments to the Authors (Required)):

The manuscript presents a haplotype-resolved diploid genome assembly for Pv11, an anhydrobiosis-capable cell line derived from the chironomid midge Polypedilum vanderplanki. The authors argue that previous genomic resources were limited by haplotype switching, consensus errors, local misassemblies, and the use of mixed insect and cell-line DNA. To address this, they generated PacBio HiFi data from a monoclonal Pv11 isolate and combined these reads with Hi-C and ONT data. The final assembly contains eight chromosome-level scaffolds. Using this assembly, the authors revisit chromosome 4, previously proposed as unusually divergent. They confirm elevated Chr4 heterozygosity with both read-based and assembly-alignment-based SNV calls, then link this divergence to gene-proximal variation, missense variants, haplotype-specific genes, and higher Ka and Ks values. Population-level analyses of independently maintained Pv11 cultures do not support ongoing Chr4-specific drift in culture. The paper also identifies two candidate telomeric repeat units, leaves centromere structure unresolved, and suggests a possible Chr2 and Chr3 fusion. Comparative synteny with Chironomus riparius indicates extensive within-chromosome gene-order reshuffling while preserving broad chromosomal identity. Finally, the authors use sBLISS to map DNA double-strand breaks during the anhydrobiosis cycle. Rehydration samples are treated cautiously because of high cell death. The main DNA damage claim is therefore based on trehalose pretreatment, where DSBs concentrate near CAGE-defined transcription start sites, accessible chromatin, and highly expressed genes.

General assessment

This manuscript provides a useful genomic resource for Pv11 cells and uses it to revisit haplotype structure, chromosome evolution, and DNA double-strand break localization during trehalose pretreatment. The strongest parts are the assembly work and the descriptive analysis of chromosome 4 heterozygosity. The sBLISS result showing promoter-proximal DSB enrichment is also convincing, especially because it is integrated with CAGE-seq and ATAC-seq.

The main weakness is that several broader biological interpretations go beyond what the current data directly test. These include the proposed causes of Chr4 divergence, the possible Chr2 and Chr3 fusion, telomere and centromere evolution, and the model that trehalose pretreatment both generates DSBs and activates protective or repair pathways. I would recommend revision before publication. The manuscript does not need to be reframed

entirely, but it should distinguish more clearly between well-supported results, plausible interpretations, and hypotheses for future work.

Specific comments

The claim that the authors have produced an improved haplotype-resolved Pv11 assembly is well supported. The authors could make the assembly evaluation more transparent in the main text. For example, a table comparing v5.2, v6.0, v6.1, v6.2, and v7.0 would help readers judge the improvement. This table should include contig number, scaffold number, total assembly size, N50 values, number of gaps, BUSCO or compleasm scores, Merqury QV, and whether each assembly is haplotype-resolved.
Most of these data were already provided in Supplementary Table S2. We have now added the number of contigs and gaps to this table.

I would suggest that the authors should avoid language that could imply a complete or near-complete assembly. The main text states that six gaps remain in chromosome-scale scaffolds and that complex terminal and centromeric regions remain unresolved. That is certainly acceptable, but the framing should consistently describe v7.0 as a high-quality draft diploid assembly rather than a complete reference.

Thank you for your suggestion. We agree with you that the term 'complete' is not appropriate for describing the v7.0 genome assembly, and we do not use it in the manuscript. Instead, we present it as a significant advancement compared to earlier versions. In the Results and Discussion sections, we also discuss several remaining limitations, including unresolved telomeres and gaps, making clear that the assembly is not complete. Conversely, the term 'draft genome' is generally used in the field to describe assemblies that are more fragmented. Therefore, we have chosen the term 'high-quality chromosome-level haplotype-resolved assembly', which is consistent with the Earth BioGenome Project assembly standards (https://www.earthbiogenome.org/report-on-assembly-standards).

To clarify this point, we have added the following text to the Discussion section:

> Despite its high base accuracy and chromosome-scale organization, the v7.0 assembly should be considered a high-quality chromosome-level haplotype-resolved assembly rather than a complete or telomere-to-telomere genome, because several chromosome-scale scaffolds retain gaps and a substantial number

of contigs remain unplaced.

The manuscript convincingly shows elevated heterozygosity on Chr4 using two independent approaches, DeepVariant on haplotype-separated HiFi reads and Syri haplotype-to-haplotype alignment. Both approaches show increased SNV density on Chr4. This makes the descriptive claim strong. The downstream claim that Chr4 divergence affects gene regions and coding sequences is moderately well supported. Chr4 shows enrichment of upstream and downstream variants, more missense variants, lower amino acid identity, more haplotype-specific genes, and elevated Ka and Ks. The expression-stratified Ka result is also consistent with stronger constraint on highly expressed genes. The interpretation that Chr4 experiences elevated mutation input or distinct selective constraints is plausible, but the current data do not separate these mechanisms. The manuscript's "and/or" phrasing is appropriate and should be retained. The authors should explicitly separate three claims: Chr4 is highly heterozygous, Chr4 variation affects gene-proximal and coding regions, and the mechanism may involve mutation rate, selection, or historical demography. The first claim is strong. The second is moderate to strong. The third remains unresolved. I would suggest that the discussion avoids implying that relaxed selection is the primary explanation unless additional tests are added. A sentence such as "These data cannot distinguish increased mutation input from altered selective constraint" would improve balance.

We have revised the Discussion subsection “Utilization of the diploid genome for evolutionary analysis” as follows:

> A parsimonious interpretation is that elevated Chr4 heterozygosity largely predates the separation of the sampled Pv11 populations and may partly reflect variation already present at or before cell-line establishment. At present, however, the available data do not distinguish among increased mutation input, altered selective constraint, and demographic effects as alternative explanations for Chr4 divergence.

The comparison with Chironomus riparius supports extensive intrachromosomal reshuffling while preserving broad chromosome identity. This claim is supported by MCScan synteny blocks and bidirectional best-hit ortholog assignments. The analysis is useful, but a two-species comparison cannot fully establish the history, rate, or direction of rearrangements. The title "Extreme genome shuffling within Chironomini" may overstate the inference. Consider changing the section title to "Extensive intrachromosomal rearrangement within Chironomini.i

The authors should also clarify whether reduced macrosynteny reflects true rearrangement, divergence affecting ortholog detection, assembly differences, or a combination. Adding

another high-quality chironomid genome, if available, would help polarize rearrangements. If no additional genome is suitable, the authors could add statistical support for synteny block depletion or rearrangement enrichment by chromosome.

We agree that the original two-species comparison could not determine whether reduced macrosynteny was specific to the Pv11–*C. riparius* comparison. We therefore extended the analysis by including *Propsilocerus akamusi*, a phylogenetically more distant species outside Chironominae. In the revised manuscript, *Pr. akamusi* is treated as Orthocladiinae sensu lato, although it has also been classified in Prodiamesinae.

Fragmented synteny was also observed in comparisons involving *Pr. akamusi*. Thus, the observed intrachromosomal reshuffling is not limited to a pairwise comparison between two Chironominae species but extends across a deeper subfamily-level divergence. We have revised the section title to "Extensive intrachromosomal rearrangement across divergent chironomid subfamily lineages" and have clarified the significance of the expanded comparison in the Results and Discussion.

We also revised the text to distinguish this supported conclusion from broader inferences that cannot yet be made. Specifically, the present three-species analysis does not allow us to reconstruct the ancestral chromosome organization, polarize individual rearrangements, or estimate rearrangement rates. We further note that sequence divergence, assembly quality, annotation differences, and ortholog-detection sensitivity may contribute to the apparent fragmentation of synteny blocks.

Minor textual suggestions

Use "extensive" rather than "extreme" for genome shuffling.
We have revised the text accordingly. The term "extreme" is no longer used in relation to genome shuffling.

Use "candidate telomeric repeats" throughout unless FISH or another localization assay is added.
Fixed accordingly.

In the abstract, reduce forward-looking claims about engineering stress-resilient cells unless that is tied more directly to demonstrated results.
Fixed accordingly.

Where the manuscript presents models, label them as models. This is especially important for the proposed link among trehalose, hyperosmotic stress, transcriptional activation, DNA repair, and promoter-proximal DSB accumulation.

The original wording could be interpreted as indicating that hyperosmotic stress directly generates DSBs and that protective or repair pathways are activated concurrently to modulate the resulting damage burden. Neither causal relationship was directly tested in the present study. We have therefore revised the Discussion to separate the observations supported by our data from the proposed mechanistic model. The revised text first states the observed enrichment of DSBs near accessible, highly expressed promoters during trehalose pretreatment and the absence of comparable promoter-proximal enrichment after H_2O_2 treatment. It then presents the relationship among promoter accessibility, transcriptional activity, and local DSB susceptibility as a working model. We also explicitly state that the causal relationships among hyperosmotic stress, chromatin state, transcription, DSB formation, and DNA repair remain unresolved and require direct functional testing.

> **The following text was added to the Discussion:**
> Collectively, our data show that trehalose pretreatment is accompanied by a non-random DSB landscape, with breaks preferentially accumulating near accessible highly expressed transcription start sites, which was not observed under the H_2O_2 exposure. These findings support a working model in which promoter accessibility and transcriptional activity influence local DSB susceptibility during trehalose-induced preparation for anhydrobiosis. However, the present study does not establish causal relationships among hyperosmotic stress, chromatin state, transcription, DSB formation, and DNA repair, which will require direct functional testing.

Reviewer #3 (Comments to the Authors (Required)):

This paper describes the sequencing, assembly and analysis of a haplotype-resolved genome of the cell line derived from the desiccation-tolerant chironomid Polypedilum vanderplanki. The authors also analysed haplotype diversity, heterozygosity patterns and haplotype-specific gene content, and suggest that specific regions are hotspots for DNA

DSBs during desiccation-inducing experiments.
While I am not an expert in all the assembly technical details, I think this is a well-written and original paper, which adds significant data to the general knowledge of desiccation, with conclusions well supported by the data.

I attach some minor comments which I suggest the Authors to address, to make the text clearer and easier to read:

Throughout the text
Some minor formatting issues, e.g.
species names not in italics (e.g. lines 75, 83, but others in the text),
Fixed accordingly

missing spaces between two words (e.g. lines 560, 690),
Fixed accordingly.

close parenthesis missing at line 768.
Fixed accordingly.

The Supplementary material was difficult to link to the text as the files names were not clear, making it difficult to find the correct file, This might be due to the submission format, but please make sure that in the final version each file is names accordingly, e.g. name "Suppl. Table 1" as such and not "4588_0_unknown_upload_747404_tcm409".
This issue should not occur when submitting a revised manuscript.

The consistent colour-coding across the figures and the very clear formatting of the Suppl. Tables make the text clear and easy to read, thank you.
We thank the reviewer for this positive comment.

I would suggest writing Latin text in italics, e.g. Latin words like in situ, versus, locus/loci, etc. We thank the reviewer for this suggestion. We have italicized Latin expressions where appropriate, including in situ. However, we have retained roman type for commonly naturalized English scientific terms, such as “versus,” “locus,” and “loci,” except where italics are required by nomenclature conventions, such as species names and gene symbols.

Abstract

Lines 21-22: Anhydrobiosis has developed in non-metazoans as well, while here the authors only mention aquatic invertebrates. While apparently a minor point, I suggest mentioning it, otherwise it looks too unique (e.g. say "in many organisms, and in some lines of aquatic invertebrates" or similar).

Fixed accordingly.

Keywords

The Authors mention sBLISS but this should be defined here, so the Keywords could be "Chironomid, Pv11 cells, anhydrobiosis, haplotype-resolved diploid genome, DNA double-strand breaks (DNA DSBs), in-suspension breaks labeling in situ and sequencing (sBLISS)".

We have removed sBLISS as it will exceed five keywords.

Introduction

Line 58, "A distinctive advantage of *P. vanderplanki* is the availability of an anhydrobiosis-capable cultured cell line, Pv11 (Nakahara et al. 2010)": I suggest including a few words about where the cell line was isolated from, just before the reference (e.g. was the cell line isolated form a single animal? Single tissue? Just to put this in context.

We have added the origin of the Pv11 cell line as follows:

> A distinctive advantage of *Po. vanderplanki* is the availability of an anhydrobiosis-capable cultured cell line, Pv11 (Nakahara et al, 2010), derived from its embryos;

Lines 120-125: if possible, I would suggest adding a figure in the text to visualise this part (the gap in Chr 4), or add this to Fig. 1A.

We apologize for the confusion. We have modified the Fig. 1 legend to clarify that the gaps are indicated in Fig. 1:

> For each chromosome, assembly gaps (vertical black lines within the gene density bar plot), HiFi read coverage (10 kb bins; black lines), and gene density (blue and green bars) are shown.

The Introduction is currently mainly focused on the genome assembly, but I would add a short paragraph on the desiccation aspects (background knowledge, the fact that desiccation can be induced by trehalose) so that the results in the section "Double-strand breaks are enriched around transcription start sites" become clearer.

We have added the following paragraph to explain anhydrobiosis in Pv11.

> Exposing Pv11 cells to 600 mM trehalose for 48 h induces anhydrobiosis-associated gene expression, enabling approximately 20% of cells to survive over 10 days of desiccation at room temperature and revive upon rehydration (Watanabe et al, 2016).

Results

At the beginning of the results, some basic summary statistics for the genome assemblies might be useful in the text (e.g. chromosome length, etc.).

We feel that listing the length of each chromosome in the main text would make the section unnecessarily lengthy. The chromosome lengths can be assessed from Fig. 1B, and additional assembly statistics are provided in Supplementary Table S2. We have added the following text to clarify that the chromosome lengths are consistent with our previously reported genome assembly:

> HiFi reads were assembled with hifiasm and integrated with previously published Hi-C data (Yoshida et al, 2022b) and ONT long reads (Miyata et al, 2022), yielding 166 haplotype-resolved raw contigs (Fig. 1A, statistics shown in Supplementary Table S2).

For reference, the sequence lengths are as follows:

Chr1_Hap1	35,171,624 bp
Chr2_Hap1	33,893,350 bp
Chr3_Hap1	30,370,807 bp
Chr4_Hap1	13,380,380 bp
Chr1_Hap2	34,988,525 bp
Chr2_Hap2	33,097,854 bp
Chr3_Hap2	30,630,506 bp
Chr4_Hap2	11,640,908 bp

Discussion

The Authors used trehalose preconditioning to trigger DNA DSBs, but this only becomes clear in the discussion I would suggest toad a short paragraph in the Introduction to make a clearer link in the Discussion.

We have added the following text in the introduction to clarify this point.

> Exposing Pv11 cells to 600 mM trehalose for 48 h induces anhydrobiosis-associated

gene expression, enabling approximately 20% of cells to survive over 10 days of desiccation at room temperature and revive upon rehydration (Watanabe et al, 2016).

It would be interesting to discuss a little bit more about horizontally transferred genes, as currently they are only mentioned in the text.

In our previous report (Yoshida et al., 2022), we identified 70 candidate horizontally transferred genes. We described these genes as follows: “Fifty of these genes were not annotated by BLASTP against Swiss-Prot using an e-value threshold of 1e-15, and the remaining 20 genes included zinc finger proteins, probable histones, lipid desaturase, and other proteins; no previously known anhydrobiosis-related genes were included in this list.” Because a detailed analysis of the HGT candidates is not the main scope of the present manuscript, we have limited our discussion to noting that the current results are consistent with our previous report on this species. We have added the following text to clarify this point:

> We also identified 128 genes with strong evidence of horizontal gene transfer (Hap1: 65; Hap2: 63; 0.41% of predicted genes), including 35 located on Chr4 (Hap1: 19; Hap2: 16; 0.11% of predicted genes). This was relatively consistent with our previous estimation of 70 horizontally transferred genes (Yoshida et al, 2022b).

Supplementary text

I would suggest integrating the Supplementary text into the main text of the paper, as some of these analyses are as interesting as the rest of the paper (and leave the Suppl Figures, Tables and their legends as supplementary material).

We thank the reviewer for this point. In our initial drafts, the material now included in the Supplementary Text was part of the main text. However, we felt that these descriptions were too detailed for the general reader and therefore moved them to the Supplementary Text. We have added the following sentences in the main text to guide readers to the relevant Supplementary Text sections if they are interested in the details.

Supplementary Text 1: Copy number variation analysis

> Window-based CNV analysis using 1-kb windows also indicated increased copy number on Chr4; however, this signal may reflect biased coverage estimates caused by the higher heterozygosity of this chromosome rather than true CNVs

(details in Supplementary Text S1; Supplementary Fig. S2D, S2E).

Supplementary Text 2: Analysis of sBLISS data from Pv11 cells

Approximately 12 DSBs per cell were detected at 193 million input reads (R03-3), where subsampling did not reach a plateau in the detection of DSBs per cell (details in Supplementary Text S2).

Supplementary Text 3: Identification of transcription start sites using CAGE-seq

To test whether these enrichments were centered on transcription start sites (TSSs), we reanalyzed published CAGE-seq data (Yamada et al, 2018) and identified 14,896 clustered transcription start sites (cTSSs), most of which were located within 250 bp of the nearest annotated start codon on the same strand (details in Supplementary Text S3; Supplementary Fig. S5).

Supplementary Text 4: Identification of open chromatin regions using ATAC-seq

A total of 16,407 ATAC-seq peaks were identified, of which 37% overlapped with the previously identified TSS positions (details in Supplementary Text S4; Supplementary Fig. S6).

We also substantially expanded the Supplementary Methods section to improve reproducibility. The revised section now describes the sBLISS workflow that was implemented in the experiment. We have also added the oligonucleotide sequences and a table linking the sample names in Supplementary Tables S9 and S10 to the SB adaptor and RPIX index of each library.

Figures

All Figures: Remove the titles e.g. "Figure 1" from each figure (as it is already in the figure legend).

Fixed accordingly.

All Figures: I realise there is lots of data in the figures, but consider making the writing larger wherever possible, to make the figures easier to read. E.g. the y axes labels of the Coverage graphs in Figure 1B could be slightly larger. Same for e.g. Figure 2, etc.

We have increased the size of the labels in the figure.

Figure 1A: the symbols for the "Unplaced contigs" is not very clear in the figure, consider making it darker/more visible.

We thank the reviewer for this point. We have chosen to remove the unplaced contigs from the genome graph, as it is not informative.

September 8, 2026

RE: Life Science Alliance Manuscript #LSA-2026-03719-TR

Prof. Takahiro Kikawada
National Agriculture and Food Research Organization
Institute of Agrobiological Sciences
Owashi 1-2
Tsukuba, Ibaraki 305-0851
Japan

Dear Dr. Kikawada,

Thank you for submitting your revised manuscript entitled "Haplotype-resolved Pv11 genome reveals chromosome evolution and anhydrobiosis-associated DNA damage". As you will see below, reviewers are satisfied with no further comments. We would be happy to publish your paper in Life Science Alliance pending final revisions necessary to meet our formatting guidelines.

MANUSCRIPT ORGANIZATION AND FORMATTING:

To avoid unnecessary delays in the acceptance and publication of your paper, please read the following information carefully. Full guidelines are available on our Instructions for Authors page, https://www.life-science-alliance.org/authors

Submission of a paper that does not conform to Life Science Alliance guidelines will delay the acceptance of your manuscript.

-Please adjust the title for a general readership who may not be familiar with the Pv11 cell line nor the term "anhydrobiosis". We welcome alternatives and would be happy to provide suggestions if you wish.
-Please remove the manuscript file provided in PDF (Previous version). We request only an editable current version of the manuscript file in .doc format. Other files you may upload with the file designation "Related manuscript file".
-Please add the X and Bluesky handles of your host institute/organization, as well as your own, and/or one of the authors, in our system.
-Please remove the number of words provided for your text and abstract on the title page of the manuscript file.
-Please rename "Competing Interest Statement" to "Conflict of Interest".
-Please add your main, supplementary figure, and table legends to the main manuscript text after the references section.
-Please add callouts for Figures 1C; S3A-C; S4B, D; S5A-C and S6B-E to your main manuscript text.
-LSA does not allow supplemental text files. Please incorporate the text and tables in this file into the Methods section and, if appropriate, the Results section. Supplemental Figure legends should be included in the main manuscript text as noted above.

We encourage our authors to provide original source data, particularly uncropped/-processed electrophoretic blots and spreadsheets for the main figures of the manuscript. If you would like to add source data, we would welcome one PDF/Excel-file per figure for this information. These files will be linked as supplementary "Source Data" files.

It is Life Science Alliance policy that if requested, original data images must be made available to the editors. Failure to provide original images upon request will result in unavoidable delays in publication. Please ensure that you have access to all original data images prior to final submission.

If you are planning a press release on your work, please inform us immediately to allow informing our production team and scheduling a release date.

We welcome submissions of potential cover images for the issue of LSA in which your work would appear. If you have high quality images associated with this work, please feel free to email these, with a caption, to the journal office.

LSA encourages authors to provide a 30-60 second video where the study is briefly explained. These videos will be appear embedded with the manuscript online at Life Science Alliance, and on social media to promote the published paper and authors (for examples, see https://docs.google.com/document/d/1-UWCfbE4pGcDdcgzcmiuJl2XMBJnxKYeqRvLLrLSo8s/edit?usp=sharing). Corresponding or first-authors are welcome to submit the video. Please submit only one video per manuscript. The video can be emailed to contact@life-science-alliance.org

FINAL FILES:

To upload the final version of your manuscript, please log in to your account: https://lsa.msubmit.net/cgi-bin/main.plex

You will be guided to complete the submission of your revised manuscript and to fill in all necessary information. Please get in touch in case you do not know or remember your login name.

The following items are required for acceptance.

-- An editable version of the final text (.DOC or .DOCX) is needed for copyediting (no PDFs).

-- High-resolution figure, supplementary figure and video files uploaded as individual files: See our detailed guidelines for preparing your production-ready images, https://www.life-science-alliance.org/authors

-- Summary blurb (enter in submission system): A short text summarizing in a single sentence the study (max. 200 characters including spaces). This text is used in conjunction with the titles of papers, hence should be informative and complementary to the title. It should describe the context and significance of the findings for a general readership; it should be written in the present tense and refer to the work in the third person. Author names should not be mentioned.

The license to publish form must be signed before your manuscript can be sent to production. A link to the license to publish form will be available to the corresponding author only. Please take a moment to check your funder requirements.

Reviews, decision letters, and point-by-point responses associated with peer-review at Life Science Alliance will be published, alongside the manuscript. If you do want to opt out of having the reviewer reports and your point-by-point responses displayed, please let us know immediately.

Thank you for your attention to these final processing requirements. Please revise and format the manuscript and upload materials as soon as you are able.

Thank you for this interesting contribution to the literature. We look forward to publishing your paper in Life Science Alliance.

Sincerely,

Tim Fessenden
Scientific Editor
Life Science Alliance

Reviewer #1 (Comments to the Authors (Required)):

Dear Authors,
Your updated manuscript concerning high-quality, haplotype-resolved genome of Pv11 cell line is well-written and solved all the points raised during the first round of review. Thank you for clarifying many points, rewriting/revising parts of Results and Discussion, especially to clearly distinguish what is directly supported by the results and what requires further validation. The additional comparisons with other species, Propsilocerus akamusi, are useful and enhance your conclusions. Also, the revised figures, including the HiC map, and supplementary materials, such as comparisons of different versions of Pv11 assemblies, are better after the review.
After reading the revised manuscript I have no further comments that require your attention, nor that could improve your manuscript.
Kind regards

Reviewer #2 (Comments to the Authors (Required)):

I am happy with the revised version of the manuscript. The authors have adequately addressed my previous comments and concerns, and I have no further issues to raise. I have no hesitation in recommending the manuscript for publication.

September 14, 2026

RE: Life Science Alliance Manuscript #LSA-2026-03719-TRR

Prof. Takahiro Kikawada
National Agriculture and Food Research Organization
Institute of Agrobiological Sciences
Owashi 1-2
Tsukuba, Ibaraki 305-0851
Japan

Dear Dr. Kikawada,

Thank you for submitting your Research Article entitled "Haplotype-resolved genome of a dry-preservable cell line reveals chromosome evolution and DNA damage". It is a pleasure to let you know that your manuscript is now accepted for publication in Life Science Alliance. Congratulations on this interesting work. We sincerely appreciate your collaboration with the editorial office to improve the title of this work so that it will be better appreciated by a broad readership.

The final published version of your manuscript will be deposited by us to PubMed Central upon publication.

Your manuscript will now progress through copyediting and proofing. It is journal policy that authors provide original data upon request.

Your article will publish open access upon publication under a CC-BY license.

Reviews, decision letters, and point-by-point responses associated with peer-review at Life Science Alliance will be published, alongside the manuscript. If you do want to opt out of having the reviewer reports and your point-by-point responses displayed, please let us know immediately.

IMPORTANT: If you will be unreachable at any time, please provide us with the email address of an alternate author. Failure to respond to routine queries may lead to unavoidable delays in publication.

Scheduling details will be available from our production department. You will receive proofs shortly before the publication date. Only essential corrections can be made at the proof stage so if there are any minor final changes you wish to make to the manuscript, please let the journal office know now.

DISTRIBUTION OF MATERIALS:
Authors are required to distribute freely any materials used in experiments published in Life Science Alliance. Authors are encouraged to deposit materials used in their studies to the appropriate repositories for distribution to researchers.

You can contact the journal office with any questions, contact@life-science-alliance.org

Again, congratulations on a very nice paper. I hope you found the review process to be constructive and are pleased with how the manuscript was handled editorially. We look forward to future exciting submissions from your lab.

Sincerely,

Tim Fessenden
Scientific Editor
Life Science Alliance
